# Functional and transcriptional profiling of non-coding RNAs in yeast reveal context-dependent phenotypes and *in trans* effects on the protein regulatory network

**DOI:** 10.1371/journal.pgen.1008761

**Published:** 2021-01-25

**Authors:** Laura Natalia Balarezo-Cisneros, Steven Parker, Marcin G. Fraczek, Soukaina Timouma, Ping Wang, Raymond T. O’Keefe, Catherine B. Millar, Daniela Delneri

**Affiliations:** 1 Manchester Institute of Biotechnology, Faculty of Biology Medicine and Health, The University of Manchester, Manchester, United Kingdom; 2 Division of Evolution and Genomic Sciences, Faculty of Biology, Medicine and Health, The University of Manchester, Manchester, United Kingdom; The Babraham Institute, UNITED KINGDOM

## Abstract

Non-coding RNAs (ncRNAs), including the more recently identified Stable Unannotated Transcripts (SUTs) and Cryptic Unstable Transcripts (CUTs), are increasingly being shown to play pivotal roles in the transcriptional and post-transcriptional regulation of genes in eukaryotes. Here, we carried out a large-scale screening of ncRNAs in *Saccharomyces cerevisiae*, and provide evidence for SUT and CUT function. Phenotypic data on 372 ncRNA deletion strains in 23 different growth conditions were collected, identifying ncRNAs responsible for significant cellular fitness changes. Transcriptome profiles were assembled for 18 haploid ncRNA deletion mutants and 2 essential ncRNA heterozygous deletants. Guided by the resulting RNA-seq data we analysed the genome-wide dysregulation of protein coding genes and non-coding transcripts. Novel functional ncRNAs, SUT125, SUT126, SUT035 and SUT532 that act *in trans* by modulating transcription factors were identified. Furthermore, we described the impact of SUTs and CUTs in modulating coding gene expression in response to different environmental conditions, regulating important biological process such as respiration (SUT125, SUT126, SUT035, SUT432), steroid biosynthesis (CUT494, SUT053, SUT468) or rRNA processing (SUT075 and snR30). Overall, these data capture and integrate the regulatory and phenotypic network of ncRNAs and protein-coding genes, providing genome-wide evidence of the impact of ncRNAs on cellular homeostasis.

## Author summary

A quarter of the yeast genome comprises non-coding RNA molecules (ncRNAs), which do not translate into proteins but are involved in the regulation of gene expression. ncRNAs can affect nearby genes by physically interfering with their transcription (*cis* mode of action), or they interact with DNA, proteins or other RNAs to regulate the expression of distant genes (*trans* mode of action). Examples of *cis*-acting ncRNAs have been broadly described, however, genome-wide studies to identify functional *trans*-acting ncRNAs involved in global gene regulation are still lacking. Here, we used a ncRNA yeast deletion collection to score ncRNA impact on cellular function in different environmental conditions. A group of 20 ncRNA deletion mutants with broad fitness diversity were selected to investigate the ncRNA effect on the protein and ncRNA expression network. We showed a high correlation between altered phenotypes and global transcriptional changes, in an environmental dependent manner. We confirmed the *trans* acting regulation of ncRNAs in the genome and their role in altering the expression of transcription factors. These findings support the notion of the involvement of ncRNAs in fine tuning cellular expression via regulation of transcription factors, as an advantageous RNA-mediated mechanism that can be fast and cost-effective for the cells.

## Introduction

Gene regulation is a key biological process across all life forms, and multiple gene interactions quickly allow adaptation to different conditions in response to environmental stimuli. This response may induce adaptation to various food sources, trigger alternative metabolic pathways, or overcome stress factors.

Chromatin modifications and DNA methylation are two main mechanisms of regulating gene expression. More recently, RNA transcripts that are not translated into protein have been described to have a prominent role as epigenetic modifiers [1, 2]. There are an increasing number of examples of these non-coding RNA (ncRNA) transcripts regulating gene expression positively and negatively [3–10].

RNA interference (RNAi) was the first understood example of ncRNA involvement in epigenetics [11]. The RNAi mechanism involves ncRNAs binding to target mRNA sequences, inhibiting their translation [12]. *Saccharomyces cerevisiae* (*S*. *cerevisiae*) lacks RNAi machinery; however, a large number of non-coding transcripts have been identified in this budding yeast using high-throughput and high-resolution technologies. These ncRNA transcripts come from what is known as “pervasive transcription”, a phenomenon that generates RNAs distinct from those that encode proteins or those with established functions (*e*.*g*. snoRNAs, snRNAs, rRNAs) [13]. Among a list of characterised pervasive transcripts, Stable Unannotated Transcripts (SUTs) and Cryptic Unstable Transcripts (CUTs) show an essential role in gene regulation, influencing histone modifications or regulating transcription of nearby genes [4, 5, 14–16].

SUTs and CUTs are polyadenylated RNAs transcribed by RNA polymerase II [17] and are distributed across the entire *S*. *cerevisiae* genome. Classically, SUTs and CUTs arise from nucleosome-depleted regions (NDRs) associated with bidirectional promoters of protein-coding genes [17, 18], but differ in their association with the RNA decay machinery. CUTs, on one hand, are degraded rapidly by the nuclear exosome and the TRAMP (Trf4-Air1/Air2-Mtr4) complex [19], whereas SUTs are only partially susceptible to Rrp6p activity [17] and are mainly affected by cytoplasmic RNA decay pathways including the translation-dependent nonsense-mediated decay (NMD) pathway and Xrn1- dependent 5’ to 3’ degradation [20]. As a result, SUTs persist longer than CUTs.

Gene regulation activities have been ascribed to SUTs and CUTs. In many cases, ncRNAs appear to cause transcriptional interference [3, 5, 15, 16, 21–23] affecting the expression of neighbouring genes in *cis*. On the other hand, ncRNAs can be functional and play a role in gene regulation in *cis* by themselves [4] or regulate in *trans* the expression of genes located both nearby or at distant loci [6, 10]. Although only a small number of functional ncRNAs have been well characterised to date, they have been shown to control gene expression at the transcriptional level. For instance, SUT075 has recently been reported to regulate the expression of *PRP3* when overexpressed remotely on a plasmid [10]. Another example is SUT457, which is involved in telomere organisation. SUT457 regulates the levels of telomeric ssDNA in a Exo1-dependent manner [9]. Interestingly, CUT281, known as *PHO84* ncRNA because it overlaps the protein-coding *PHO84* gene, triggers *PHO84* silencing in a *trans* and *cis* manner using two independent mechanisms. While the *cis-*acting mechanism requires Hda1/2/3 deacetylation machinery, *trans* function depends on the Set1 histone methyltransferase [5, 6].

Emerging evidence has suggested that ncRNAs play roles in the recruitment of transcription factors (TFs) to their binding sites in fission yeast, mouse and humans [21–26], thus, suggesting a conserved mechanism of gene expression among eukaryotes. On one hand, ncRNA expression around regulatory elements can locally promote TF binding [23, 24]. On the other hand, ncRNA can regulate gene expression by acting as binding competitors for DNA-binding proteins (DBPs) [25, 26].

Considerable progress has been made over the past decade to elucidate the unique features and molecular mechanisms of ncRNAs. However, detailed insights have been limited to single ncRNA genes, usually affecting neighbouring genes. Here, we combine large-scale phenotypic analysis with RNA-seq technology to generate a global view of the transcriptome following ncRNA deletion. Specifically, by analysing the expression network, we show that the global transcriptional effects of deleting four SUTs individually are indirect and act via specific TFs whose level of expression is affected by deleting these ncRNAs. This *trans* effect supports and extends previous premises that SUTs or CUTs are a functional part of the genome and can influence the general transcriptional output of a cell independent from where they are located.

## Results and discussion

### Fitness profiling of haploid ncRNA deletion strains reveals plasticity of phenotypes in different environmental conditions

**Fig 1:**
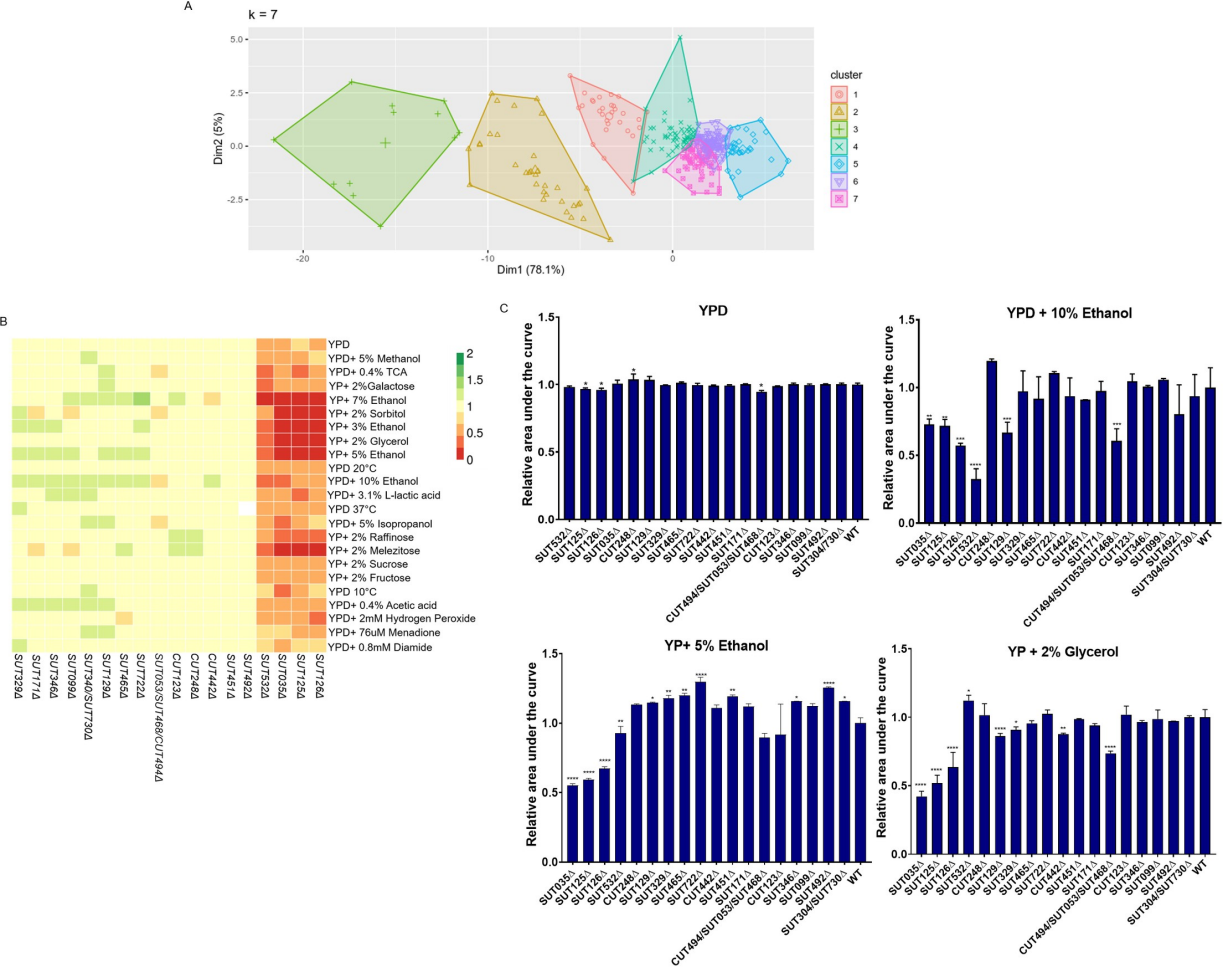
Principal component analysis and fitness profile of the ncRNAs deletion strains in solid and liquid media. (A) Principal Component Analysis (PCA) for the fitness profile of 372 ncRNAs deletion strains. The seven functional clusters identified, highlighted in different colour, correspond to the deletion strains with similar phenotypes in 23 different environmental conditions. (B) Heat-map of the 18 haploid ncRNA deletion strains analysed in this study. Rows represent the different growth conditions and columns represent the ncRNAs. Colour bars represent the colony size normalised to the wild-type strain which is given the arbitrary growth value of 1. Fitness reduction, increase, and no change is represented as shades of red, green, and yellow, respectively. Missing data is represented as white. (C) Liquid growth assays for the 18 haploid ncRNA deletion strains analysed in this study. Bar charts show the relative area under the curve for haploid SUT and CUT deletion strains grown in YPD, YPD +10% ethanol, YP + 5% ethanol and YP +2% glycerol. The data are presented as means calculated from three biological replicates normalised to WT. Comparisons between wild-type and mutants were analysed using ANOVA followed by Dunnett’s test.

To investigate the plasticity of organism fitness in response to ncRNA deletions, we acquired phenotypic data for the haploid ncRNA deletion collection generated by Parker *et al* [10] in 23 different conditions. The ability of 50 CUT, 93 SUT, 61 snoRNA and 168 tRNA deletion mutant strains to utilise different carbon sources, and to tolerate extreme pH and oxidative stress was scored. The colony size was used as a proxy for fitness and normalised to the wild-type strain per condition according to Tong and Boone [27]. The ncRNA deletion mutants showing similar behaviour across the 23 different conditions were grouped, generating 7 distinct functional clusters (Fig 1A). The list of deletion mutant strains in each cluster is reported in the Supplementary S1 Dataset. A complete clustered heatmap showing individual phenotypes of ncRNA deletions as a pdf vector graphic is shown in S1 File.

About 45% of the ncRNA deletion mutants analysed did not show significant phenotypic changes in any condition tested. The deletion of 25% of the tRNAs tested affected fitness, whereas a much larger amount, namely 62%, of the snoRNA tested displayed a significant impact on the fitness compared with WT (S1 Dataset, S1 File). The effect of the snoRNA deletions were predominantly under oxidative stress conditions such as in media containing Menadione, Hydroxide Peroxide or Diamide. This was somewhat expected given that box C/D snoRNAs regulate the response to oxidative stress pathways in mammalian cells [28, 29]. Despite the lack of a clear assigned cellular function, 29% of SUTs and 22% of CUTs tested altered the cell phenotype, either showing fitness improvement or defect when deleted in some environmental conditions. Of the SUTs that showed alteration in fitness, a subset of four, namely SUT125, SUT126, SUT035, SUT532, displayed the most severe defects in the majority of the conditions, especially when grown in ethanol, glycerol, sorbitol, galactose or melezitose as carbon sources (Fig 1B).

The conditions in which the least number of ncRNA deletion mutants were affected were YP+ 2% fructose, YPD + 5% methanol, and YPD + 5% isopropanol, which affected 4.3%, 4.5% and 5.1% of ncRNA deletion mutants, respectively. Conditions that induced the broadest fitness changes were YP + 7% ethanol and YP + 2% glycerol, with 11.8% and 11.5% of ncRNA deletion mutants affected, respectively (S1 Dataset).

Liquid growth assays were set up for a selection of SUTs and CUTs mutants that displayed different phenotypic fitness profiles in solid media (Fig 1B): SUT125, SUT126, SUT035, SUT532, with severe fitness defect; CUT494/SUT053/SUT468, SUT099, SUT171 with mild or moderate fitness defect; SUT129, CUT123, SUT465, CUT248, SUT722, SUT304/730, SUT329, SUT346, showing increased fitness; and CUT442, SUT451, SUT492 showing mostly no phenotypic changes. The liquid growth phenotype was normalised against the WT and is reported as relative area under the growth curve (Fig 1C). A breakdown for the different growth phases is presented in S1 Table. Overall, in rich media, the majority of deletion mutant strains showed no growth differences, with the exception of the reduced fitness of *SUT125*Δ, *SUT126*Δ and *CUT494/SUT053/SUT468Δ* and the improved fitness of *CUT248Δ* (Fig 1C and S1 Table).

When 10% ethanol was added to the media, *SUT125Δ*, *SUT126Δ*, *SUT035Δ*, *SUT532Δ*, *SUT129Δ* and *CUT494/SUT053/SUT468Δ* displayed severe fitness defects, affecting the majority of the growth phases (Fig 1C and S1 Table); a similar profile for fitness impairment for SUT125, SUT126 and SUT035 was observed in media containing either 5% ethanol or 2% glycerol (Fig 1C). However, *SUT532Δ* and *SUT129Δ* had a divergent fitness profile in 5% ethanol and 2% glycerol. *SUT532Δ* presented a significant fitness defect in YP + 5% ethanol and a growth improvement in YP+ 2% glycerol (Fig 1C), whereas *SUT129Δ* showed an improvement in YP + 5% ethanol and defect in 2% glycerol.

Several SUTs and CUTs displayed improved fitness in the YP + 5% ethanol liquid media (Fig 1C) revealing a similar phenotypic change in both solid and in liquid media. About 56% of the strains grown in YPD +10% ethanol and 27.7% of the strains grown in YP + 2% glycerol displayed some differences in fitness profiles between solid and liquid media. For example, *CUT494/SUT053/SUT468Δ*, *SUT129Δ*, *SUT329Δ* and *CUT442Δ* exhibited fitness impairment in YP + 2% glycerol which was not previously detected in the solid fitness assay. Discrepancies between solid and liquid fitness are likely due to the differing oxygen availability and diffusion rates of one or more nutrients on solid media [30–35]. Indeed, when growing on solid surfaces, colony morphology differs between yeast growth phases and time [35–37]. Therefore, these results re-iterate the importance of acquiring data from both solid and liquid growth assays for an accurate picture of cellular fitness.

### ncRNA deletions drive global transcriptional changes that correlate with phenotypic profiles

The main function previously ascribed to ncRNAs in budding yeast was transcriptional regulation, usually of neighbouring or overlapping single genes [3–5, 7, 16, 38]. We therefore investigated by RNA-seq whether selected ncRNA deletion mutants with altered phenotypes also had dysregulated transcriptomes. We selected 18 haploid ncRNA deletion mutants from clusters 3,4,5 and 6 with different types of phenotypic changes (*i*.*e*. growth defects, improvements and no changes) to study by RNA-seq, together with heterozygous deletions of 2 essential ncRNAs, namely SUT075 and snR30 (Table 1; please note that SUT075 is a partial deletion previously described in Parker *et al* [10]). To identify the genes that are differentially expressed (DE) in the mutant strains, a 1.5-fold-change (FC) and two different q-value cut offs, <0.05 and <0.1, were used. We obtained the same overall trend of significant results using either q-value (S2 Dataset). For further analysis we chose to use the less stringent cut off of <0.1 to increase the probability of detecting relatively minor effects on genes, especially on neighbouring genes, and of identifying biological pathways that might be compromised by the deletion of the ncRNAs. As expected, we detected changes in the levels of at least one neighbouring transcript in 8 of the ncRNA deletion mutant strains analysed by RNA-seq. Three of these deletion mutants (*SUT099Δ*, *SUT722Δ*, *SUT171Δ*) up-regulate only their neighbouring genes, while the remainder (*CUT494/SUT053/SUT468Δ*, *SUT532Δ*, *SUT035Δ*, and *SUT125Δ*) also revealed altered levels of distantly located transcripts. Strikingly, over one-third of the deletion mutants studied by RNA-seq had large numbers (>100) of DE coding and non-coding transcripts (Table 1).

**Table 1:** Numbers of protein-coding genes and non-coding transcripts that are differentially expressed (q-value cut off < 0.1 and absolute fold change ≥1.5) in 18 haploid SUT and CUT deletants and 2 heterozygous deletions. A ‘neighbour’ gene or transcript is defined as an adjacent genomic feature.

	Protein- coding genes				Non-coding DNA			
SUT/CUT	Number of DE genes	Up -Regulated	Down-Regulated	Neighbour DE gene	Number of DE transcripts	Up -Regulated	Down-Regulated	Neighbour DE transcript
SUT035	700	256	444	1	232	138	94	0
SUT125	721	310	411	2	196	106	90	0
SUT126	787	335	452	0	223	141	82	0
SUT532	408	236	172	0	92	55	37	1
CUT494/SUT053/SUT468	137	102	35	1	32	6	26	0
CUT123	0	0	0	0	0	0	0	0
CUT248	2	2	0	0	0	0	0	0
SUT129	16	8	8	0	2	1	1	0
SUT304/ SUT730	1	0	1	0	0	0	0	0
SUT329	1	0	1	0	0	0	0	0
SUT722	1	1	0	1	0	0	0	0
SUT171	2	1	1	1	0	0	0	0
SUT346	1	0	1	0	0	0	0	0
SUT465	0	0	0	0	0	0	0	0
CUT442	2	0	2	0	0	0	0	0
SUT099	2	1	1	1	0	0	0	0
SUT451	0	0	0	0	0	0	0	0
SUT492	1	0	1	0	0	0	0	0
snR30*	2276	1063	1213	0	408	335	73	2
SUT075*	2284	1057	1227	1	292	238	54	0

* *snR30* and *SUT075* essential ncRNA.

Altered RNA levels could be a consequence of either transcriptional or post-transcriptional changes. However, we saw no difference between WT and mutant strains in the relative distribution of reads across DE ORFs or their 3’UTRS (S2 File), arguing against differences in post-transcriptional RNA processing. Additionally, none of the genes with significant changes in RNA level contain introns, hence altered splicing does not appear a possibility here. Therefore, these results point to transcriptional changes as the primary mechanism for the detected alteration of RNA levels.

Half of the deletion mutant strains had smaller numbers of differentially expressed transcripts, while only three deletion mutants did not lead to transcriptional changes in rich medium. Overall, transcription profiles of the ncRNA deletion mutants correlated well with their fitness changes. For instance, heterozygous deletions of the two essential ncRNAs SUT075 and snR30 have a stronger negative effect on strain fitness in all the conditions tested (S1 Fig). As expected, these deletions affected the largest number of transcripts (Table 1).

The two apparently unrelated essential ncRNAs, SUT075 and snR30, have a surprisingly large number of DE transcripts in common (about 80%; 864 up-regulated and 972 down-regulated). Gene Ontology (GO) analysis of the shared DE protein-coding genes revealed enrichment for ribosome biogenesis, ribosomal RNA processing, DNA replication and the cell cycle (S2 Fig). This GO enrichment is consistent with the known role of snR30 in ribosomal RNA processing [39]. SUT075 is required for normal transcript levels of its neighbouring essential gene *PRP3* and can act in *trans* [10]. We note however, in our RNA-seq data, that the down-regulation of *PRP3* was not significantly strong (FC, 0.7) in *SUT075*Δ deletion and instead a large global effect on the transcriptome was detected, including targets in common with the snR30 mutant. A further 481 essential genes are affected in addition to *PRP3* when SUT075 is partially deleted (82 up-regulated and 399 down-regulated) representing 43% of the *S*. *cerevisiae* essential genes. As a comparison, *snR30*Δ dysregulates 450 essential genes (ca. 40%), up-regulating and down-regulating 82 and 368, respectively. Nineteen small RNAs are dysregulated in *snR30*Δ, including the essential snR19 and LSR1 (U1 and U2 snRNAs) that are part of the major spliceosome in yeast, and the RNA component of nuclear RNase P (*RPR1*) and RNase MRP (*NME1*). Interestingly, 17 snoRNAs, of which 11 are in common with *snR30*Δ, are also differentially expressed in *SUT075*Δ. Our data suggest that several factors, including an effect on the neighbouring gene *PRP3* and a potential role in rRNA processing, may cause the essentiality of SUT075.

### Phenotypic and transcriptional effects on neighbouring genes of the *KanMX* cassette used to generate ncRNA deletions

**Fig 2:**
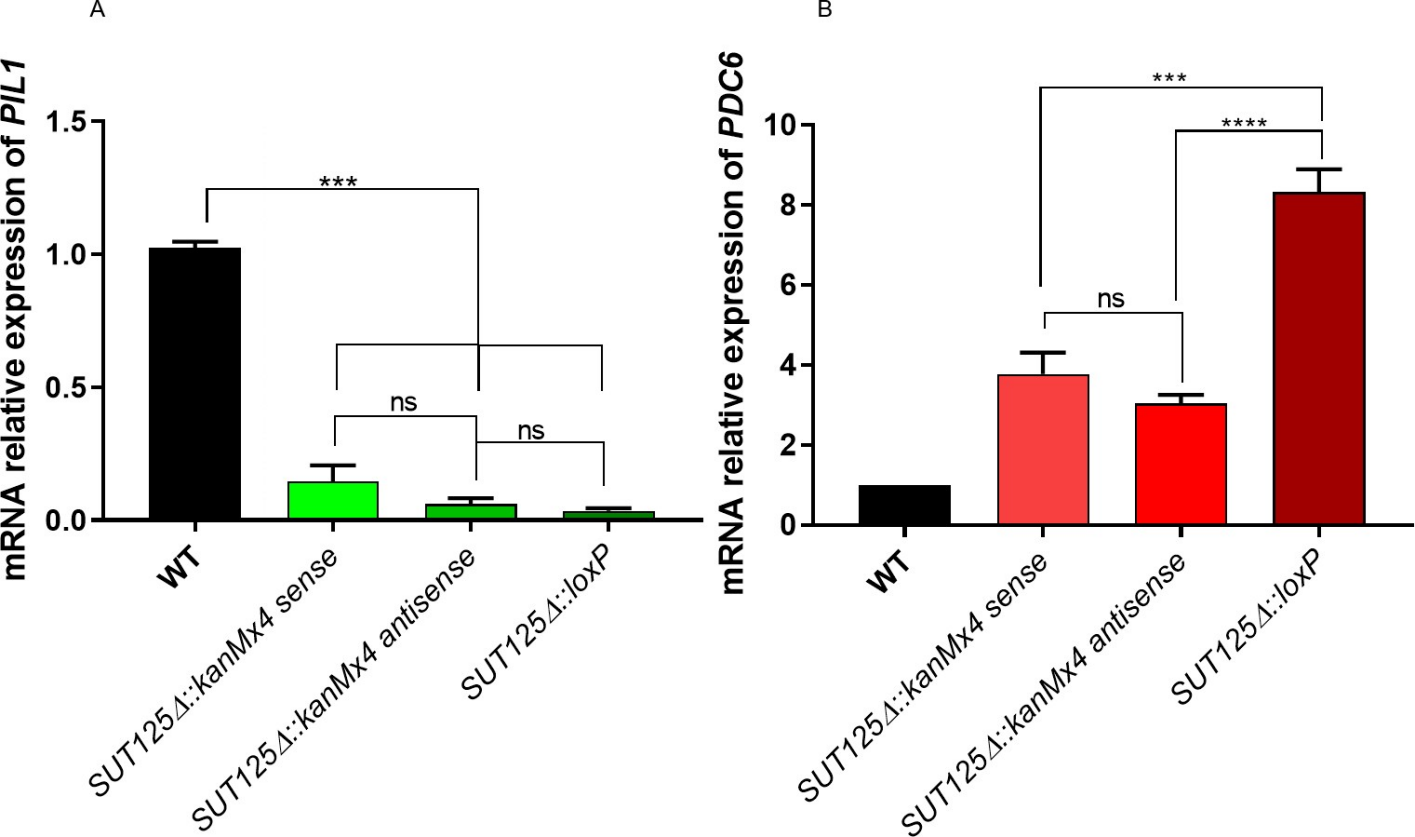
Actively transcribed *kanMX* partially decreases the regulatory effect of neighbouring genes in *SUT125* deletion strains. Transcriptional changes of SUT125 neighbouring genes (A) *PIL1* and (B) *PDC6* in *SUT125*Δ mutant strains with sense, antisense orientations (relative to SUT125) of the *kanMX* cassette, and without *kanMX* after excision with the Cre/loxP system. Relative mRNA levels were quantified by qPCR and compared by *ANOVA* followed up to Tukey-Kramer test.

The *kanMX* cassette used to make the ncRNA deletion mutant strains has been suggested to affect the expression of neighbouring genes, either because of its high transcriptional level or via the generation of unexpected antisense transcripts [40–43]. We did not observe any alteration in transcript levels of neighbouring genes in the majority (13/20) of the ncRNA deletion mutant strains that we studied (Table 1) but levels of one or both neighbouring transcripts were affected in the remainder and might, therefore, contribute to the observed changes in phenotype and gene expression. For example, *SUT125Δ*, besides globally affecting the transcriptome, also had an effect on both of its neighbouring genes, *PIL1* and *PDC6*. Levels of *PIL1 mRNA* are reduced while *PDC6* transcript levels are higher in the mutant (S3 Fig and S2 Dataset). To test whether *kanMX* cassette insertion replacing SUT125 causes the local expression changes, the mRNA levels of *PDC6* and *PIL1* were quantified and compared in three different SUT125 deletion mutant strains containing: *i*. the *kanMX* cassette in sense orientation relative to *SUT125*; *ii*. the *kanMX* cassette in antisense orientation relative to *SUT125*; *iii*. a *loxP* scar after *kanMX* excision with the Cre/loxP system (i.e. no *kanMX* cassette). The down-regulation of the expression of *PIL1* remains the same in all three mutants, ruling out a transcriptional effect of the *kanMX* cassette on *PIL1* expression (Fig 2A). *PDC6* is up-regulated in all three mutants, however the effect is stronger when the *kanMX* cassette is removed (Fig 2B). This result suggests a partial effect of the *kanMX* cassette on *PDC6* expression, where the presence of the cassette either in sense or antisense orientation dampens the up-regulatory effect.

**Fig 3:**
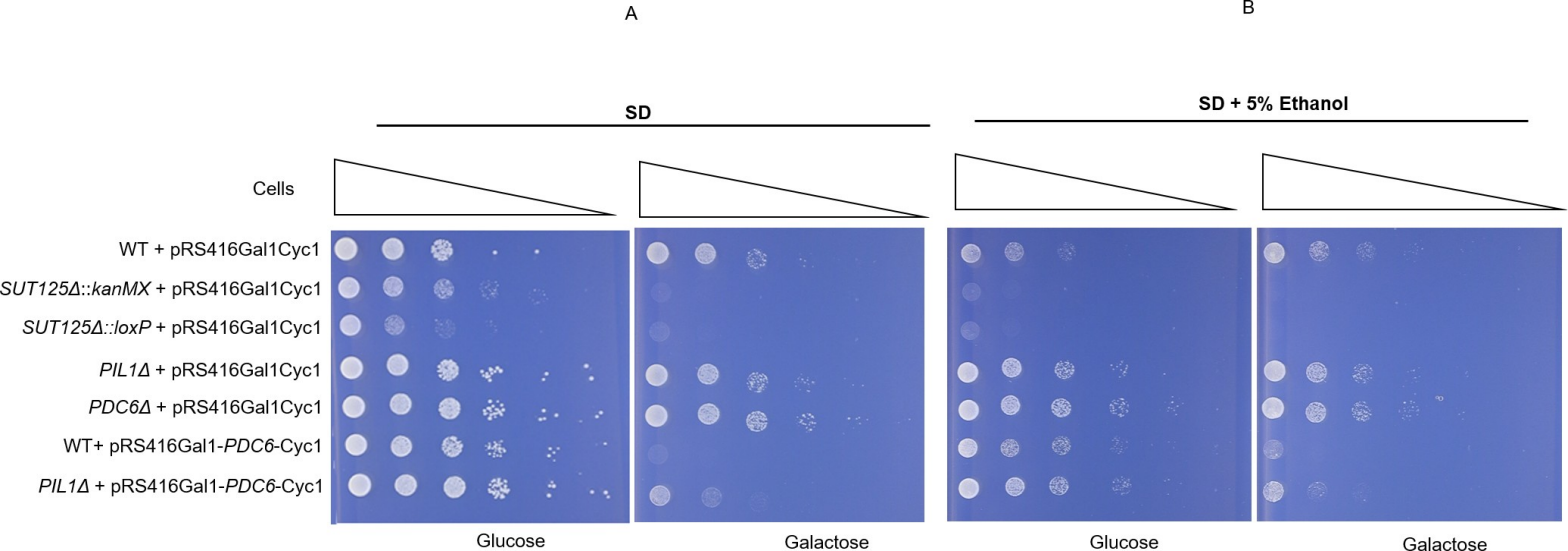
*PDC6* overexpression may explain the majority of the *SUT125Δ* phenotype. Spot test assay of: *SUT125Δ* deletion strains with and without *kanMX; PIL1Δ* deletion strain; *PDC6* overexpression strain; and *PIL1Δ* with *PDC6* overexpression plasmid plated on (A) Synthetic minimal medium lacking uracil (SD-Ura) and (B) SD-Ura + 5% ethanol, containing either 2% glucose or 2% galactose as indicated below each panel. The *PDC6* overexpression plasmid has the *PDC6* gene under control of the inducible *GAL1* promoter in the pRS416 plasmid. Wild-type and deletion strains containing the pRS416Gal1Cyc1 (empty plasmid) and the *PDC6Δ* deletion strain were included as controls.

To identify whether *PIL1* down-regulation and *PDC6* overexpression trigger the growth changes observed in *SUT125Δ* in medium containing ethanol, we carried out spot test growth assays. *PDC6* was overexpressed from a plasmid to mimic up-regulation, and a *PIL1* deletion strain was used to mimic *PIL1* downregulation. The combined effect was scored in a *PIL1Δ* strain harbouring the *PDC6* overexpression plasmid. Presence or absence of the *kanMX* cassette reveals little to no effect on the resulting phenotype (Fig 3). Overexpression of *PDC6* in a WT background produced the same phenotype as a SUT125 deletion, while either *PIL1Δ* or *PDC6Δ* deletion did not have any effect on the phenotype (Fig 3). The concomitant effect of over-expressing of *PDC6* in *PIL1Δ* strain produced a less severe, but still comparable, phenotype to that of a SUT125 deletion. These data suggest that *PDC6* overexpression alone may account for the majority of the phenotype following *SUT125Δ* deletion. Growth impairment under non-fermentable carbon sources is not surprising when *PDC6* expression is altered, because of its role in alcoholic fermentation, committing pyruvate to ethanol production rather than the TCA cycle and aerobic respiration [44, 45].

**Fig 4:**
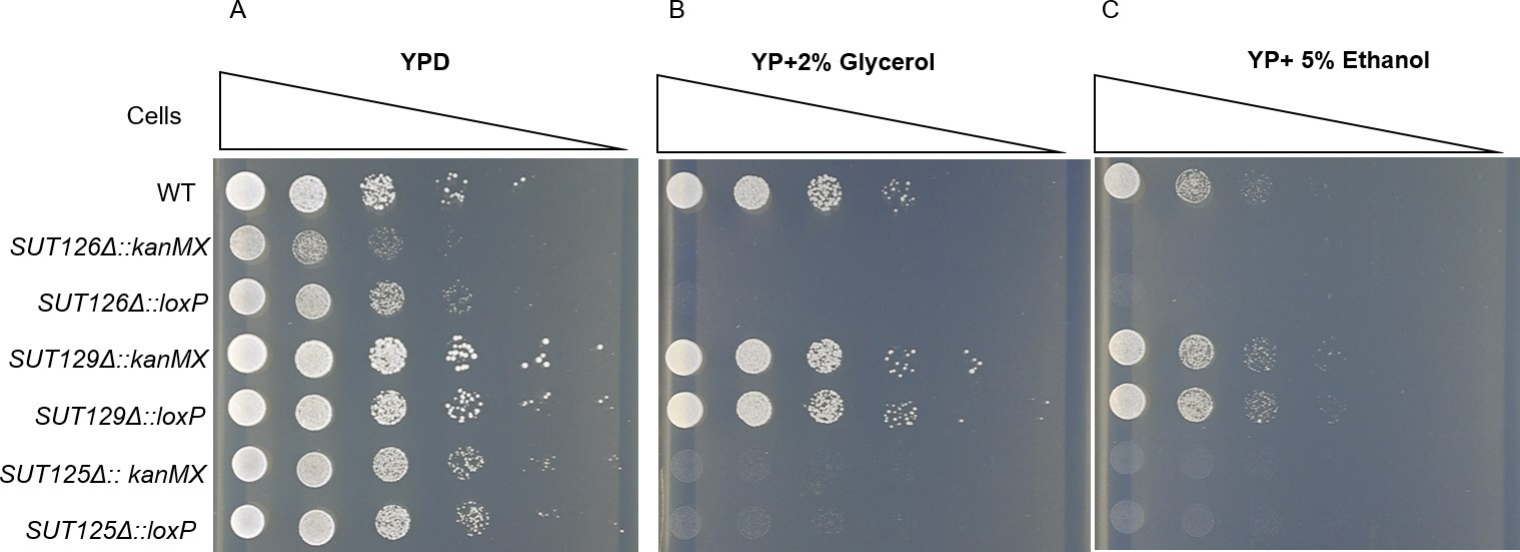
Presence or absence of *the kanMX* cassette does not affect growth phenotypes in ncRNA deletion strains. Spot test assay of: BY4741 (WT), *SUT125Δ SUT126Δ* and *SUT129Δ* with and without *kanMX* on (A) YPD; (B) YP + 2% glycerol; and (C) YP + 5% ethanol.

The effect of the *kanMX* cassette on growth phenotypes was also tested in *SUT125Δ* and *SUT126Δ*, which has a fitness impairment, and *SUT129Δ*, which displays a fitness gain. Similar fitness profiles were observed regardless of the presence or absence of *kanMX* for all the ncRNA mutants (Fig 4). In addition, the effect of the *kanMX* selectable marker on the transcription of distant DE genes was tested. The mRNA level of two transcription factors, *PDR3* and *YOX1*, differentially expressed in *SUT125Δ* and *SUT126Δ* (S2 Dataset) was quantified via qPCR in the same mutants with and without *kanMX*. No significant differences in the expression levels of *YOX1 and PDR3* were detected (S4 Fig). In summary, these data indicate that phenotypic and transcriptional changes observed in these ncRNA deletion mutants are not dependent on the presence of an actively transcribed drug resistance marker gene.

### ncRNAs SUT125, SUT126, SUT035 and SUT532 act in *trans* to regulate target genes

**Fig 5:**
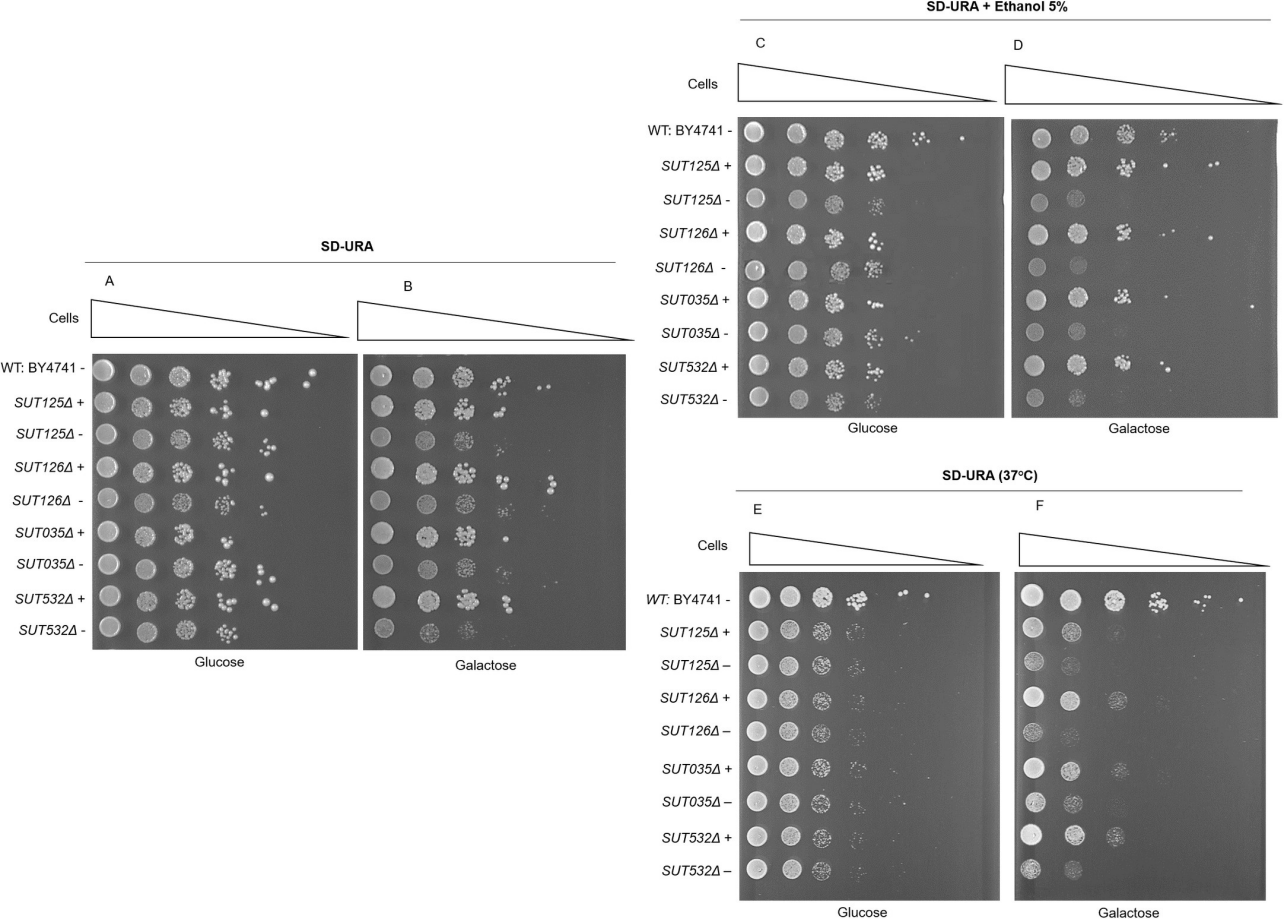
SUTs whose deletion results in global transcriptional changes can rescue growth phenotypes in *trans*. Rescue spot test analysis of growth phenotypes of ncRNA deletion mutant strains. The indicated SUT under control of the *GAL1* promoter, was spotted at 30°C onto SD + 2% glucose (A), SD + 2% galactose (B), SD + 2% glucose and 5% ethanol (C) and SD + 2% galactose and 5% ethanol (D). Additionally, the spot assay was carried out at 37°C in SD + 2% glucose (E) and SD + 2% galactose (F). +: pRS416 with the respective SUT;—: empty plasmid.

The deletion of SUT125, SUT126, SUT035 or SUT532 led to extensive changes in the global transcription network (S2 Dataset) and large fitness defects (Fig 1B). Based on the fitness profile of those ncRNAs, we assessed the ability of SUT125, SUT126, SUT035 and SUT532 to ectopically rescue growth in galactose and in the presence of additional stresses such as ethanol and high temperature (Fig 5). Each of these SUTs was placed under control of an inducible *GAL1* promoter on a plasmid that was transformed into the respective deletion mutant. Under conditions where the *GAL1* promoter is repressed (glucose) there were no significant differences in growth between deletion strains carrying the *GAL1-SUT* plasmid or the *GAL1* plasmid without SUT. When *GAL1*-driven expression was induced, all four SUTs were able to rescue the small growth defect of the mutants in SD-galactose (Fig 5A and 5B) and the larger defect in SD-galactose with 5% ethanol (Fig 5C and 5D); while a partial rescue was scored in SD-galactose at 37°C (Fig 5E and 5F). The rescue is, therefore, dependent on the plasmid that is carrying the particular ncRNA. These results suggest that SUT125, SUT126, SUT035 and SUT532 can act *in trans*, which may underlie the altered regulation of large numbers of genes in these mutants. There are only a handful of examples of *trans* acting ncRNAs in yeast but CUT281, that can act both in *cis* and *trans* to repress the *PHO84* gene [5, 6], and SUT457 which act in *trans* to rescue the phenotype of telomeric overhang accumulation observed in *SUT457Δ* cells [9].

### Discordant changes between transcriptome and fitness as a tool to reveal additional context-dependent phenotypes

Generally, ncRNA deletion mutants that show large fitness defect also produce significant global transcriptional changes (*i*.*e*. *SUT035Δ*, *SUT125Δ*, *SUT126Δ* and *SUT532Δ*), and vice versa. All CUTs analysed displayed modest phenotypes and did not show any effect on the transcriptome (or only few genes), with the exception of CUT494/SUT053/SUT468 that, surprisingly, dysregulated a large number of transcripts (over 150). To investigate this apparent discrepancy, we used GO analysis to identify enriched functional categories across the 137 DE genes in *CUT494/SUT053/SUT468Δ*. The majority of the over-represented GO terms were related to the synthesis of crucial membrane components and membrane fluidity pathways. Specifically, GO biological process categories enriched among down-regulated genes included sterol, steroid, ergosterol and lipid biosynthesis, while up-regulated genes were clustered in pathways for propionate metabolism, drug response and molecular transport (S5 Fig). Ergosterol (ERG) is an essential membrane component that regulates membrane fluidity, permeability, membrane-bound enzyme activity and substance transportation [46]. Overexpression or deletion of ERG biosynthesis genes results in the accumulation of toxic intermediates, alteration of drug sensitivity and slow growth in different media, including non-fermentable carbon sources [47]. Interestingly, our fitness data revealed a growth defect of the *CUT494/SUT053/SUT468*Δ strain in YP + 2% Glycerol (Fig 1C). To test the hypothesis that *CUT494/SUT053/SUT468*Δ has a role in membrane stability by targeting synthesis of ERG, we tested this deletant strain alongside the WT and the other three CUTs mutants, in the presence of azole antifungal agents that inhibit various steps in the ERG biosynthesis pathway [48]. Since *CUT494/SUT053/SUT468Δ* is also affecting the expression of its neighbouring gene *MRH1*, we have also included the *MRH1Δ* deletion strain in the growth assay to rule out that the potential phenotypic effects are due to *MRH1* dysregulation. *MRH1* is an integral membrane protein, with unknown function, with its expression reduced upon *CUT494/SUT053/SUT468Δ* deletion.

**Fig 6:**
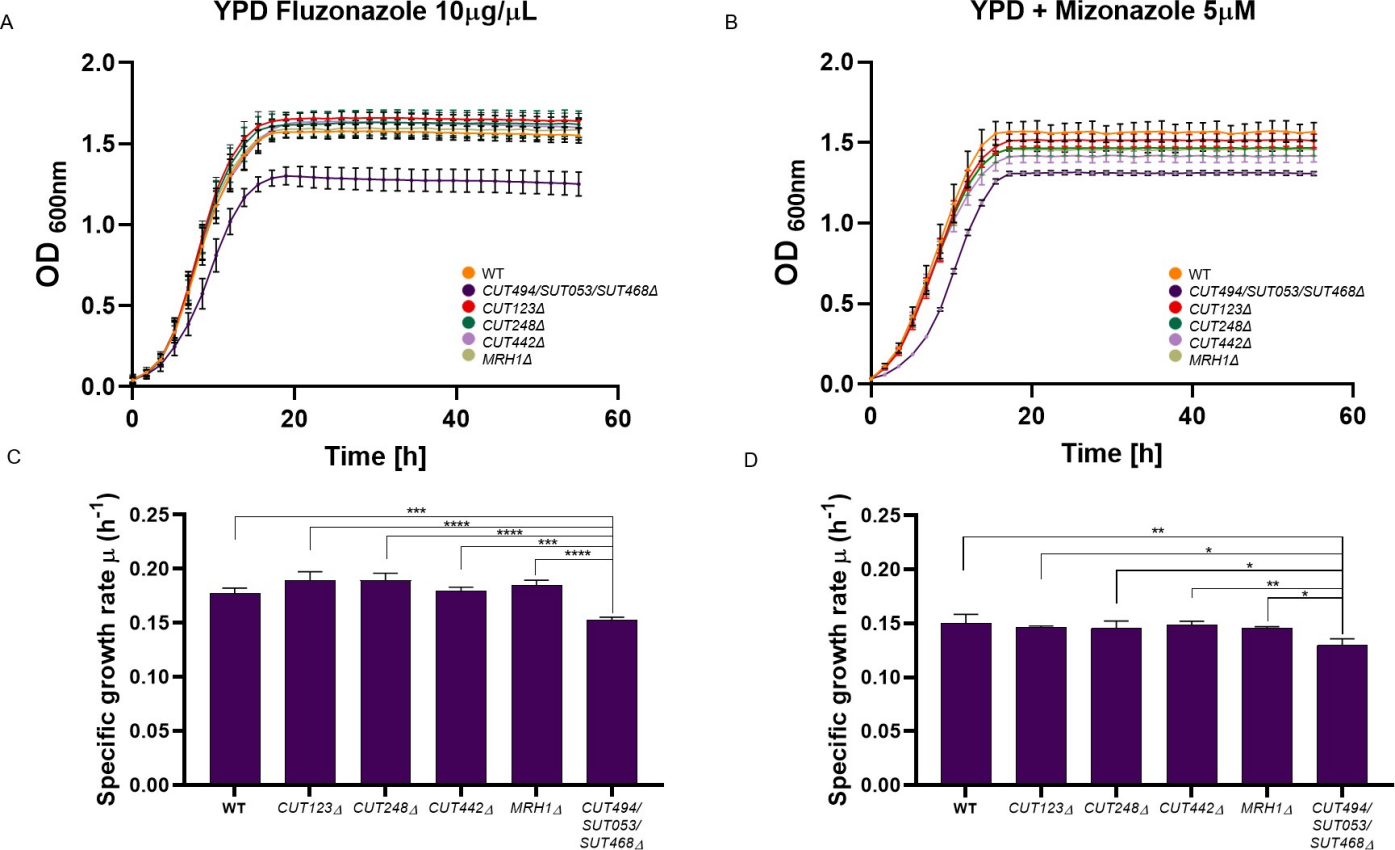
Liquid growth assays of ncRNA deletion mutants in the presence of azoles. Growth curves of *CUT123Δ*, *CUT248Δ*, *CUT442Δ*, *CUT494/SUT053/SUT468Δ*, *MRH1Δ* and WT strains in YPD media supplemented with (A) fluconazole (10 μg/μL) and (B) miconazole (5 μM). Bar charts show the mean specific growth rate (μ) of WT and ncRNA deletion strains in the presence of (C) fluconazole (10 μg/μL) and D) miconazole (5 μM). Significance of differences was assessed by *ANOVA* followed up to Tukey-Kramer test.

When the fitness of *CUT494/SUT053/SUT468Δ* was tested in medium supplemented with either fluconazole (Fig 6A and 6C) or miconazole (Fig 6B and 6D), a slow growth phenotype was identified for *CUT494/SUT053/SUT468Δ* compared to the WT, while *MRH1Δ* and the other CUT deletion mutants display a growth similar to the WT (Fig 6).

Additionally, when *CUT494/SUT053/SUT468Δ* growth is compared with *MRH1Δ* in YPD supplemented with 10% ethanol, or with media containing non-fermentable carbon sources (*i*.*e*. glycerol or ethanol) *MRH1* does not display the same fitness impairment as *CUT494/SUT053/SUT468Δ* (S6 Fig). These data confirm that the phenotype detected is due to the effect *CUT494/SUT053/SUT468* deletion has on the expression of distant genes. This result also suggests that transcriptome data can be used to identify environmental conditions that are likely to reveal fitness defects.

### ncRNAs with related phenotypes regulate common genes involved in mitochondrial functions

**Fig 7:**
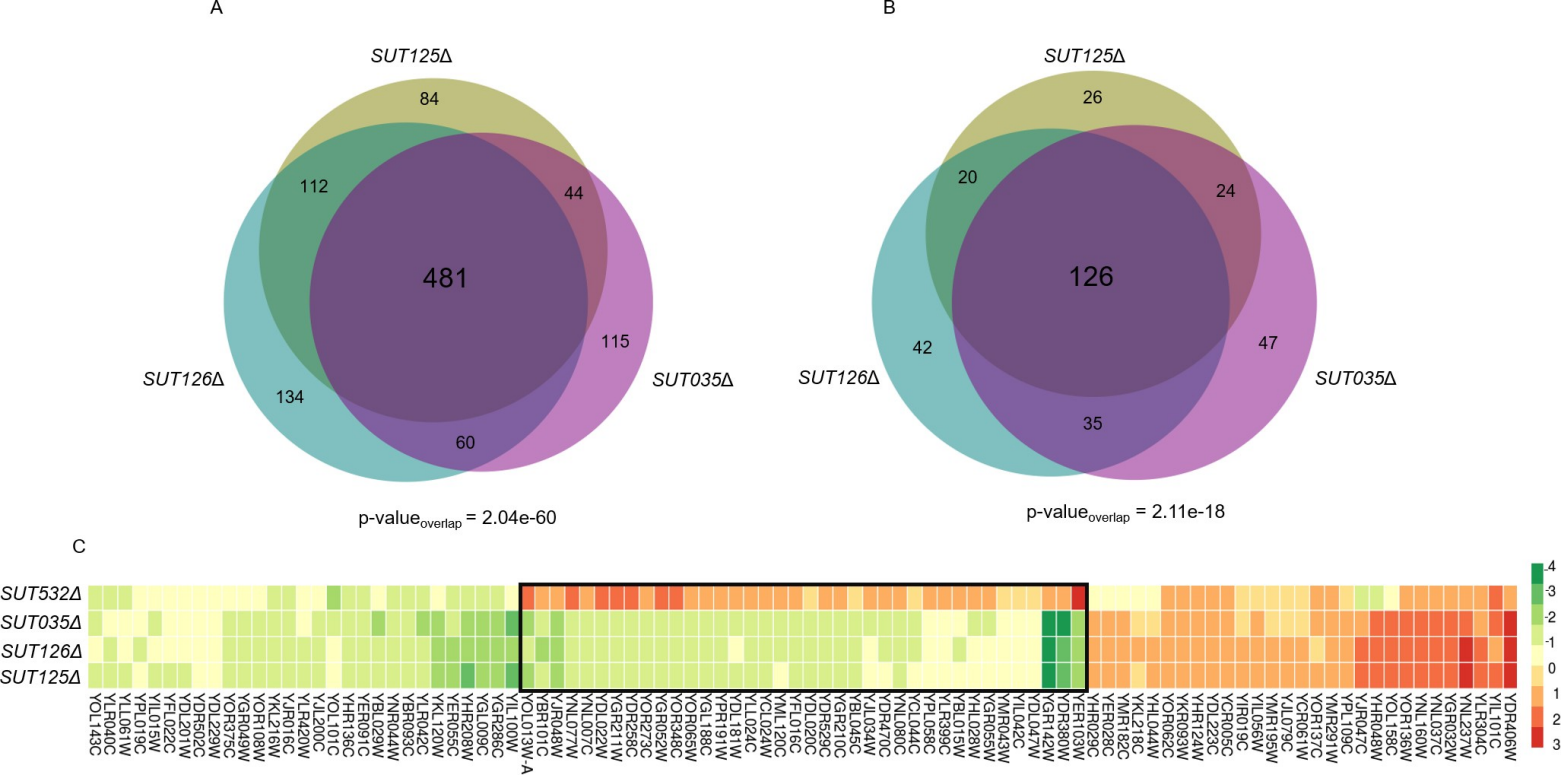
Deletion mutant strains displaying identical fitness profiles share a significant number of differentially expressed coding and non-coding transcripts. Area proportional Venn diagrams displaying the number of differentially expressed (A) Protein-coding genes (intersection p-value = 2.04e-60 and (B) Non-coding transcripts (intersection p-value = 2.11e-18) in common between *SUT125Δ*, *SUT035Δ* and *SUT126Δ*. Venn diagrams were generated with BioVenn [90]. (C) Heat map of differentially expressed genes in common between ncRNA deletion mutants with similar fitness profiles. Heat map was constructed with 96 common DE genes between *SUT125Δ*, *SUT126Δ*, *SUT035Δ* and *SUT532Δ*. Colours represent the change in expression of genes, as indicated in the key on the right. DE genes in *SUT532Δ* with different transcriptional directionality from the other three ncRNA deletants are boxed.

SUTs/CUTs clustered together by their fitness profile are expected to engage similar biological and molecular functions. To test this premise, we identified the set of common DE genes across deletion mutant strains that are part of the same phenotypic cluster. Remarkably, SUT125, SUT126 and SUT035 when deleted dysregulated 481 coding genes (286 downregulated and 195 up-regulated) and 126 non-coding transcripts in common (Fig 7A and 7B). Moreover, those ncRNA deletion mutants displayed negative fitness during phenotypic analysis when grown in 22 out of the 23 media tested (S1 File). To demonstrate the accuracy of our gene expression measurements, we selected a few candidate DE genes from the SUT125, SUT126 and SUT035 deletion mutants, and tested their mRNA levels by RT-qPCR. Among the selected genes, down-regulated and up-regulated expression fold change by qPCR were similar to the expression fold change obtained from the RNA-Seq data (S3 Fig and S2 Dataset), validating the RNA-seq data.

To identify the biological processes associated with the commonly misregulated genes in SUT125, SUT126 and SUT035 the set of DE genes was analysed for GO term enrichment and the most significant hits were selected. Genes with decreased and increased expression were associated with key mitochondrial functions such as mitochondrial electron transport and oxidation-reduction process (S7 Fig). The enriched pathways identified from KEGG and Reactome data (Holm-Bonferroni correction) were branched amino acid biosynthesis (p-value 5e-4 7 matches), aerobic respiration, electron transport chain (p-value = 0.002 11 matches), mevalonate pathway (p-value = 0.004 5 matches) and TCA cycle (p-value = 0.021 9 matches), also indicating roles in mitochondrial functions. When SUT532 is included along with SUT125, SUT126 and SUT035, there are 96 protein-coding genes and 15 non-coding genes dysregulated in common (S8 Fig). Those common genes have, in general, a concordant expression profile between each ncRNA deletion mutant strain. However, for 40% of the common genes, specifically those involved in mitochondrial function, an opposite expression trend is detected in the *SUT532*Δ strain compared to *SUT125*Δ, *SUT126*Δ and *SUT035*Δ. Since the phenotypes of *SUT125*Δ, *SUT126*Δ, *SUT035*Δ and *SUT532*Δ mutants in different environmental conditions are mostly similar, except in glycerol; these 18 genes with different directionality of expression may either not be crucial for the observed phenotype, or specific to the mechanism of action for SUT532 in the cell (Fig 7C). Due to the divergent fitness shown in glycerol for *SUT532*Δ strain, we sought to elucidate if there are specific mitochondrial pathways in which SUT532 could be involved. Thus, GO of the non-common genes (321) for this ncRNA deletion mutant was also performed. Interestingly, up-regulated genes are related to the TCA cycle and aerobic respiration along with protein refolding and response to stress. Down-regulated genes are mainly involved with leucine biosynthesis biological process (S9 Fig). Taken together, these results reveal enrichment of mitochondrial roles for SUT125, SUT126, SUT035 and SUT532, suggesting their potential function in repressing or activating mitochondrial metabolic pathways, justifying the fitness impairment of those deletion mutants when grown with non-fermentative carbon sources.

### ncRNAs drive global transcriptome changes through transcription factors

The finding that large numbers of genes involved in the same pathways are DE in different ncRNA deletion mutant strains suggests that these ncRNAs may be acting via sequence-specific TFs that regulate these groups of genes. Dysregulation of similar transcripts might also be related to a stress response induced by the absence of such transcripts. Evidence of different non-lethal stresses were shown to induce a common coordinated transcriptional stress response which entails the down-regulation of ribosomal genes, genes involved in RNA metabolism, protein synthesis, and the up-regulation of genes controlled by TFs involved in the general stress response, such as Msn2 and Msn4 [49–51]. Using the YEASTRACT database [52–55], we identified TFs that are up- or down-regulated in *SUT125*Δ, *SUT126*Δ, *SUT035*Δ, *SUT532*Δ and *CUT494/SUT053/SUT468*Δ mutants, which all show large transcriptional changes. We found that several TFs were significantly perturbed in *SUT125*Δ, *SUT126*Δ, *SUT035*Δ, *SUT532*Δ and *CUT494/SUT053/SUT468*Δ, affecting ca 16%, 19%, 20%, 13% and 5% of all annotated yeast TFs (ca. 183), respectively. In *CUT494/SUT053/SUT468*Δ, *SUT126*Δ, *SUT035*Δ, and *SUT532*Δ the number of TFs with altered expression is significantly higher than expected by chance when comparing the total number of DE genes and the total number of TFs (p-values < 0.05; S2 Table). Several DE TFs, such as *PDR3*, *MOT3* and *YOX1*, were shared among *SUT125Δ*, *SUT126Δ*, *SUT035Δ* (S10 Fig). The expression changes for these three TFs were validated with *SUT126Δ* via real time PCR (S3 Fig), showing a strong agreement between the qPCR and RNA seq data.

As the most significant fitness phenotypes observed for ncRNA deletion mutant strains were in YP or YPD media supplemented with ethanol, we identified those TFs whose mis-regulation has been linked to ethanol resistance. Many ethanol-tolerance genes share a TF-binding motif recognised by Pdr1 and Pdr3 [56]. In the *S*. *cerevisiae* genome, 12.39% of genes are Pdr3 targets [55]. Strikingly, about 95% (*p* < 0.0001) of DE genes in *SUT126*Δ, *SUT125*Δ and *SUT035*Δ are targets of this zinc finger protein that acts predominantly as a transcriptional activator [55, 57, 58] and whose mRNA levels significantly increase in the same ncRNA deletion mutant strains (S2 Dataset and S3E Fig). Furthermore, Msn4, a key regulator for ethanol and stress response [55, 59], is up-regulated when SUT532 is deleted and down-regulated in *SUT035Δ* deletion strain (S2 Dataset); however, causing mainly a similar phenotype in different stress conditions (Fig 1B and 1C). Accordingly, 40.4% of dysregulated genes in the *SUT532Δ* and 37.7% in *SUT035Δ* are targets of Msn4. It is not surprising that different *MSN4* modulation caused similar effect on fitness. In fact, it is known that overexpression or downregulation of *MSN4* can lead to either mild or severe fitness defect [51, 60], like the one we observed in both *SUT532Δ* and *SUT035Δ* deletion strains (Fig 1B and 1C). These data suggest that SUT125, SUT126, SUT035 and SUT532 ncRNAs are associated with mechanisms of ethanol tolerance and stress response that may involve a massive gene expression reprogramming resulting from the shift from fermentative to non-fermentative metabolism. Moreover, they imply that ncRNAs may be part of the activation or repression of metabolic pathways and regulatory networks through modulation of TFs.

**Fig 8:**
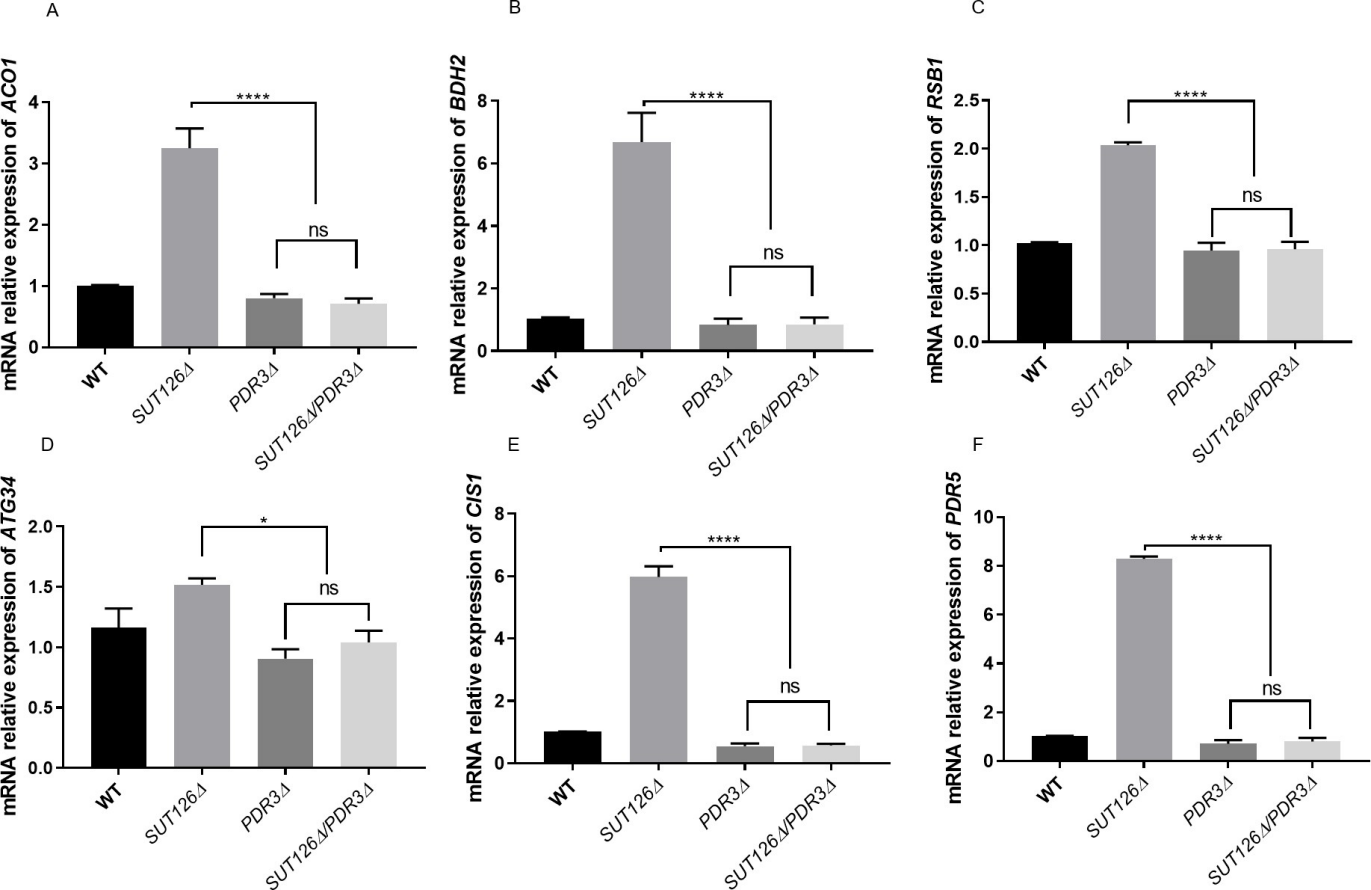
Indirect gene expression changes may be partially driven by SUT126 ncRNA acting through transcription factors. Relative mRNA levels of (A) *ACO1*, (B) *BDH2*, (C) *RSB1*, (D) *ATG34*, (E) *CIS1*, and (F) *PDR5* analysed by RT-qPCR with *SUT126Δ/PDR3Δ* single and double mutants. The increased levels of *PDR3* targets in the *SUT126Δ* single mutant are dependent on Pdr3 (*ANOVA*).

To test whether the upregulation of Pdr3 target genes upon SUT126 deletion is Pdr3-dependent, we investigated the expression of previously validated Pdr3 target genes [57, 61–71] in the *SUT126Δ* background. We found that the SUT126 deletion is not sufficient to activate Pdr3 target genes *ACO1*, *BDH2*, PDR5, *CIS1*, *ATG34* and *RSB1* in the absence of Pdr3 (Fig 8). These results suggest that the global effect on the transcriptome observed in the absence of SUT126 is likely driven by an effect of this ncRNA on TFs such as Pdr3. SUT126 may have a repressive effect on the promoter of *PDR3*, may destabilise the *PDR3* transcript, or, as *PDR3* is autoregulated, may bind to and interfere with the Pdr3 protein.

**Fig 9:**
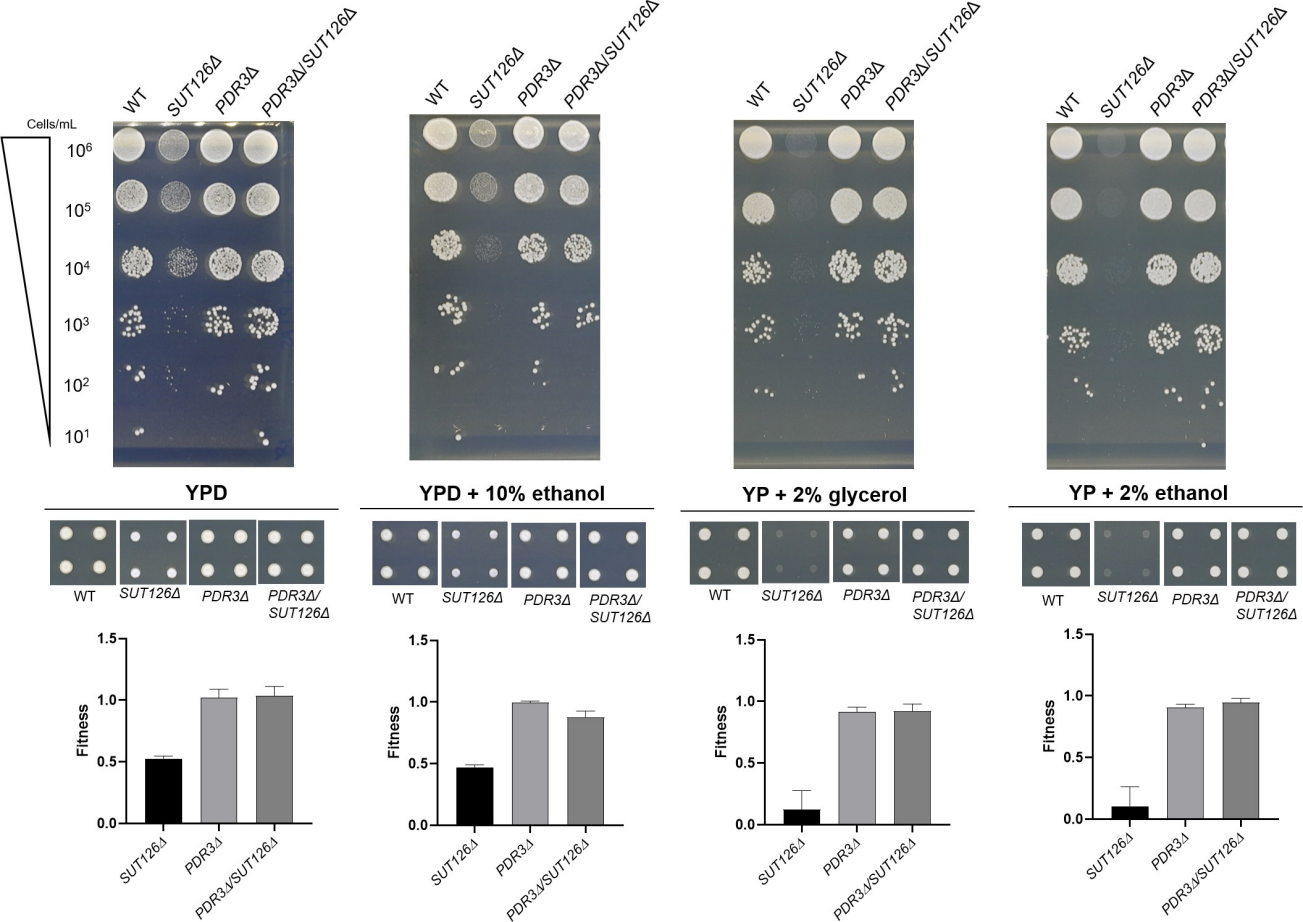
SUT126 display a positive genetic epistasis with Pdr3. Spot test assays were carried out for *SUT126Δ*, *PDR3Δ*, *PDR3/SUT126Δ* and WT on YPD, YPD + 10% ethanol, YP + 2% glycerol and YP + 2% ethanol. The normalised colony size was used as proxy to calculate the fitness of the single and double mutants represented in the bar charts.

To look for genetic interactions between Pdr3 and SUT126, we tested the growth of the single *PDR3Δ* and *SUT126Δ* strains against the double mutant *PDR3Δ/SUT126Δ* in four media: YPD, YPD + 10% ethanol, YP + 2% glycerol and YP + 2% ethanol. We confirmed that *PDR3* and *SUT126* show positive epistasis in all conditions since the deletion of *PDR3* in a *SUT126Δ* background was able to rescue the growth defect observed in *SUT126Δ* mutant (Fig 9; S3 Table). These results show that SUT126 phenotype is primarily *PDR3* dependent. The data also show that while the deletion of *PDR3* has little effect on the phenotype, the overexpression of this TF, upon SUT126 deletion (Fig 9), has a large negative effect on cellular growth in the condition tested.

Several ncRNAs have been reported to bind TFs to regulate gene expression in other organisms. For example, in mice, the long-ncRNA (lncRNA) *linc-YY1*, involved in myogenesis, has been found to interact with the TF *YY1* [24]. Similarly, *GAS5* interacts with glucocorticoid receptors, supressing their binding with glucorticoid response elements [26]. In humans, lncRNA rhabdomyosarcoma 2–associated transcript (RMST) interacts directly with Sox2, a transcription factor involved in the regulation of embryonic development [72]. Regulation of gene expression by ncRNAs acting through transcription factors might, therefore, be a conserved mechanism among eukaryotes. In this way, ncRNAs could confer an extra advantage to yeast cells by modulating gene expression in response to environmental stress.

## Conclusion

Large-scale phenotypic projects using deletion mutant collections have proven to be an invaluable tool for linking genes to their function [73–77]. Here we used 372 haploid strains from the ncRNA deletion collection [10, 76] to identify deletions that are responsible for phenotypic changes in 23 environmental conditions. The fitness data obtained has been integrated into the Yeast ncRNA Analysis (YNCA) database (http://sgjlab.org:3838/ynca/) [10]. Based on the phenotypic screening data, we further analysed 20 ncRNA deletion mutants at the transcriptome level. ncRNA deletion mutants that were phenotypically impaired also triggered significant changes in the gene regulatory network. By analysing the expression data, we identified specific pathways where these SUTs and CUTs were functioning, such as mitochondrial function and respiration, ethanol tolerance, rRNA processing, plasma-membrane fluidity and sterol biosynthesis. In the *SUT126*Δ strain, we showed that the large transcriptional changes are due to the altered expression of TFs rather than the direct effect of the lncRNA deletion. These results indicate that ncRNAs are likely to be involved in fine tuning expression by regulating the expression of TFs.

Gene regulation driven by ncRNAs through TFs may be a conserved mechanism amongst eukaryotes. Examples of ncRNAs enhancing the loading of TFs at their target promoters or acting as a binding competitors for DNA/RNA binding proteins in fission yeast, mouse and human cells are increasing [23–26]. In fact, most ncRNAs are transcribed near regulatory units for transcription such as promoters or enhancers [17, 18, 78], which may be an indication that associates them with biological function and mechanism.

We discovered that SUT125, SUT126, SUT532 and SUT035 act in *trans* since their functions can be rescued ectopically. Strikingly, these ncRNAs originate from intergenic regions that do not overlap with any open reading frame, bearing out the possibility that their functionality may be linked with their potential to form accessible structural domains able to bind to DNA, RNA or proteins [79–81]

Such ncRNA mediated regulation is cost-effective compared to the classical regulation via TFs as the fast production of RNAs compared to proteins facilitates quick genetic responses to environmental stimuli.

## Materials and methods

### Yeast strains, growth conditions and plasmids

A list of *Saccharomyces cerevisiae* strains and plasmids is provided in S4 Table. For strain maintenance and construction, strains were grown at 30°C under standard conditions. ncRNA single deletion strains used this study were taken from the ncRNA deletion collection created by Parker *et al* [10, 78]. Deletion mutants were maintained on Yeast extract Peptone Dextrose Agar (YPDA) containing 200 μg/mL G418. Double deletion mutant strains were constructed by substituting the candidate *SUT* locus with the *natNT2* cassette and were maintained on YPDA containing 100 μg/mL clonNAT.

For construction of strains ectopically expressing particular SUTs, isogenic wild-type and ncRNA deletion mutant strains cells were transformed with pRS416-Gal1-Cyc1 overexpression plasmid containing the ncRNA of interest. Resulting strains were maintained in a synthetic minimal media lacking uracil (SD-Ura: 1X Yeast Nitrogen Base (YNB) (Formedium); 1X Complete Supplement Mixture (CSM)–Ura (Formedium); 2% (w/v) glucose). For phenotypic rescue studies, strains were grown to an optical density at 600 nm (OD_600_) of 0.5 in YP (1% yeast extract, 2% peptone) medium supplemented with 2% raffinose (YPRaf) at 30°C and induced with YP medium containing 2% galactose (YPGal) for 2 hours before being harvested for spot test assays.

### Cre recombinase-mediated marker excision in *Saccharomyces cerevisiae*

SUT deletion strains containing loxP sites flanking the *kanMx* cassette were transformed with pSH-ble^r^ plasmid DNA, and grown on YPDA containing 10 μg/mL phleomycin. To excise the cassette, cells harboring pSH-ble^r^ were grown overnight in YPRaf medium, re-suspended in 10 ml YPGal medium to an OD_600_ of 0.3 and incubated at 30°C for 3 h. The culture was diluted and plated out on YPDA. The resulting colonies were replica-plated on YPDA containing 200 μg/mL G418 to confirm the marker loss and YPDA with 10 μg/mL phleomycin to confirm the plasmid loss. The marker loss was also verified by colony PCR.

### Phenotypic analysis on solid and liquid media

Two biological and four technical replicates of the haploid deletion mutant strains were arrayed in 384 well microtitre plates. Using a Singer Rotor HDA, the 384 well cell cultures were stamped onto YPDA plates and replica plated onto 23 different environmental conditions and incubated at a particular temperature. A full list of the media and temperatures used in this study are listed in S6 Table. Plates were imaged at 24, 48 and 72 hours using a Bio-Rad Gel Doc XR system and images were processed using SGAtools [82]. The initial normalisation was performed in the software in order to correct for uneven plate growth according to the plate median and row/column correction. Minimum and maximum spot sizes were set to 0.02 and 100, respectively. The average pixel count for the biological replicates of each strain were then normalised to the appropriate plate wild-type value. Mean, standard deviation and p-values were calculated assuming a normal distribution of values. The colony sizes were then standardised to make each condition comparable (S11 Fig).

The optimal number of clusters were optimised by Gap Statistic model (S11C Fig) [83] and K- means algorithm was used as clustering method [84] Deletion strains with similar growth pattern in different media were grouped into 7 specific clusters.

For genetic interactions single and double mutant SGA plates were digitally photographed in white light using the Phenobooth (Singer Instruments) and colony areas were obtained and process using SGAtools. The genetic interaction (SGA score; ε) was scored by comparing the single mutant fitness (*W*_*A*_, *W*_*B*_) and double mutant fitness (*W*_*AB*_) derived from normalized colony size measurements; $\varepsilon =W_{AB}-W_{A}\;x\;W_{B}‐$ [85] Absolute SGA score |ε| > 0.08, and p-value < 0.05 were used as a defined confidence threshold for significant interactions.

For spot test assays, cultures were grown overnight before being serially diluted 1:10 and spotted onto agar plates.

For liquid fitness assays, cells were grown at 30°C from an OD_600_ nm of 0.1, and growth measurements at OD595nm were recorded using a BMG FLUOstar OPTIMA Microplate Reader. The readings were taken every 5 minutes as previously described by Naseeb and Delneri [86] for up to 55 hours incubation time. Three technical replicates of three independent biological samples were used for each deletion mutant and wild-type strain. Graphs and growth parameters were produced using the *grofit* package of the *R* program.

### Total RNA extraction and quantitative RT-PCR

Total RNA was isolated from 1x10^7^ cells using the RNeasy Mini Kit (QIAGEN, Germany) following the protocol for enzymatic digestion of cell wall followed by lysis of spheroplasts. To eliminate genomic DNA contamination, an additional DNAse treatment was performed with RNAse-free DNase set (QIAGEN, Germany) following the manufacturer’s protocol. The RNA extracted was quantified using a NanoDrop LiTE Spectrophotometer (THERMO SCIENTIFIC, United States). Two micrograms of total RNA were reverse transcribed into cDNA using SuperScript III Reverse Transcriptase (Invitrogen, UK) according to the manufacturer’s protocol. Optimised qPCR reactions contained 2ng/μl of cDNA, 3pmol each primer and 5 μL of iTAq Universal SYBR Green super Mix 2X in a final volume of 10 μL. Reactions were cycled on a Roche Light Cycler real time System for 35 cycles of: 15 seconds at 95°C; 30 seconds at 57°C; and 30 seconds at 72°C. Three biological replicates and three technical replicates per sample were used in each experiment, and all runs included a no template control, and a control lacking reverse transcriptase. The relative expression of each gene was estimated using the Ct values relative to those of *ACT1*. Primers were designed to produce an amplicon of 80–150 bp (Sequences given in S5 Table).

### Illumina HiSeq library preparation and sequencing

Libraries were prepared from total RNA using the TruSeq Stranded mRNA Library Prep Kit (Illumina,Inc) according to the manufacturer’s instructions. Sequencing was performed on an Illumina HiSeq4000 instrument. Sequences corresponding to protein-coding genes were mapped to sacCer3, while CUT and SUT sequences were mapped using the genomic coordinates provided by Xu *et al* [17]. Mapping was performed using STAR [87]. Differential gene expression analysis was based on the negative binomial distribution (DESeq2) [88] and a q-value cut off of <0.05 and <0.1 were applied (S2 Dataset). Genes with a statistically significant difference in expression from wild-type, as indicated by a q-value below 0.1, and greater than 1.5-fold change in expression, were used for subsequent analysis.

### Bioinfomatic and statistical analyses

Differentially expressed genes were listed and grouped as up- or down-regulated. Enriched GO terms and pathways were identified using YeastMine, with the Helmed- Bonferroni correction used to calculate adjusted *p*-values [89]. The Yeast Search for Transcriptional Regulators And Consensus Tracking (YEASTRACT) [55] database was used to look for transcription factors and their target genes.

Defective 3’end processing was assessed by visualising the distribution of reads across the gene body and at the at the 3’-end of the top 20 DE genes in each mutant strain from the RNA-seq experiments. Read density was normalized per gene to take in account the difference in expression between WT and mutant strains (S2 File).

Statistical tests were performed using Welch two sample t-test and multiple comparisons were analysed using ANOVA followed by Dunnett’s test or Tukey-Kramer test, according to the experiment. Error bars denote standard deviations except where noted and *p*-values are indicated on Figs as: * p < 0.05 ** p < 0.01 ***p < 0.001 ****p <0.*0001;* ns = no significant change.

## Supporting information

S1 Dataset This file contains the solid fitness data of the 372 mutant strains tested in 23 different conditions. Mutant strains are grouped per clusters.
(XLSX)

S2 Dataset This file contains the RNA-seq data divided by mutant, including the list of significant DE genes per mutant strain. Tables are divided by protein-coding genes and non-coding transcripts.
(XLSX)

S1 File This file contains a clustered heatmap showing individual fitness profile of 372 ncRNA deletions in 23 different environmental conditions. Colour bar represent the normalised colony size. Fitness reduction is represented as shades of red. Fitness increased is represented as shades of green. No fitness change is represented as yellow. Missing data is represented as white. The description for all the Condition (1–23) is listed in S6 Table.
(SVG)

S2 File This file contains a succession of images showing the RNAseq reads across the 20 top differential expressed genes in *SUT125Δ*, *SUT126Δ*, *SUT035Δ* and *SUT532Δ* mutant strains. The entire ORF and a specific “zoom in” on the 3’UTR is visualised.
(DOCX)

S1 Fig Solid fitness of heterozygous deletions of essential ncRNAs, SUT075 and snR30. Bar charts displays the colony size of *SUT075Δ* and *snR30Δ* deletion strains when growing in (A) YPD and (B) YPD supplemented with 10% ethanol.
(TIF)

S2 Fig Gene ontology for biological process enriched in DE genes in common between snR30 and SUT075. Bar chart displaying the top 20 significantly enriched GO terms. The negative logarithm of the adjusted p-value (base 10) after Holm-Bonferroni correction is represented on the x-axis. The figure was created using the DE genes in common for SUT075 and snR30 deletion mutants (n = 1836).
(TIF)

S3 Fig Validation of DE genes obtained during RNA-seq by qPCRs. Relative mRNA levels of (A) *PDR3* and (B) *YOX1* in *SUT035*Δ strain, (C) *PDC6* and (D) *PIL1* in *SUT125*Δ and the TFs (E) *PDR3*, (F) Y*OX1* and (G) *MOT3* in *SUT126*Δ strain analysed by RT-qPCR. Relative mRNA levels were quantified by qPCR and compared by *t*-test.
(TIF)

S4 Fig Altered expression levels of target genes in ncRNA deletion mutant strains are independent of *kanMX* marker. Relative mRNA levels of the transcriptional repressor (A) *YOX1* and the transcriptional activator (B) *PDR3* in *SUT125*Δ and *SUT126*Δ deletion mutant strains with and without *kanMX*. The *kanMX* cassette does not influence genes located distantly from the SUT disruption. Relative mRNA levels were quantified by qPCR and compared by *ANOVA*.
(TIF)

S5 Fig Histogram of GO terms from DE genes in *CUT494/SUT053/SUT468Δ* strain. Representative GO terms for biological processes for up-regulated (red) and down- regulated (green) genes in the *CUT494/SUT053/SUT468Δ* strain. The p-value cutoff after Holm-Bonferroni correction is < 0.05; y–axis displays GO terms, x-axis shows the p-value that was transformed to–log10. The figure was created using the DE genes (n = 137).
(TIF)

S6 Fig Comparison between deletion phenotypes of CUT494/SUT053/SUT468 and its neighboring *MRH1* gene. The growth of *CUT494/SUT053/SUT468Δ*, *MRH1Δ* and WT strains were analysed via spot tests on YPD, YPD + 10% ethanol, YP + 2% glycerol, YP + 2% ethanol and YPD+ 10μg/μL fluconazole. *MRH1* is an integral component of the membrane, downregulated in *CUT494/SUT053/SUT468Δ* mutant strains, and located nearby. The fitness impairment detected in *CUT494/SUT053/SUT468Δ* strain under stress conditions is independent of its effect on the neighboring gene *MRH1*.
(TIF)

S7 Fig SUT125, SUT126 and SUT035 reveal an important role in mitochondrial processes. Gene Ontology of biological processes inferred from dysregulated coding targets in common in *SUT125*Δ, *SUT126*Δ and *SUT035*Δ deletion strains. Significantly first enriched GO terms for biological processes (Holm-Bonferroni adjusted p-value <0.05) are listed on the y-axis, and the negative log of the adjusted p-value (base 10) is represented on the x-axis. The figure was created using the DE genes in common for SUT125, SUT126 and SUT035 (n = 481).
(TIF)

S8 Fig Area proportional Venn diagram of DE transcripts between SUT125, SUT126, SUT035 and SUT532. Number of (A) protein coding genes (96) and (B) non-coding transcripts (15) in common dysregulated among *SUT125Δ*, *SUT035Δ*, *SUT126Δ* and *SUT532Δ* deletion strains. Venn diagram was generated using Eulerr [91].
(TIF)

S9 Fig Gene Ontology of biological processes inferred from DE protein coding genes in S*UT532Δ* deletion mutant strain. Significantly enriched representative GO terms for biological processes for up-regulated (red, n = 172) and down-regulated (green, n = 236) in *SUT532Δ* deletion strain. P-value was calculated using Holm-Bonferroni correction. Representative GO terms are listed on the y-axis, and the negative log of the adjusted p-value (base 10) is represented on the x-axis.
(TIF)

S10 Fig Venn diagram representing TFs in common between phenotypic related ncRNA deletion mutants with significant impact on the genome. Area proportional Venn diagram generated by BioVenn [71] using the number of TFs dysregulated in deletion strains in cluster 1 (*SUT125*Δ, *SUT035*Δ) and 2 (*SUT126*Δ). The overlapping (23 TFs) is shown in a dark green colour.
(TIF)

S11 Fig Normalisation and optimal number of clusters calculated from the phenotypic data of ncRNAs deletion strains. Representative box plot of raw (A) and normalized (B) data from three technical replicates of colony size for 372 ncRNAs deletion strains in 23 different environmental conditions. The box plot indicates median-centered raw data distributions of the fitness per condition tested, which were further refined during normalisation. The horizontal axis stands for the media, while the vertical axis represents the colony sizes. (C) Optimal number of clusters were calculated by the Gap Statistic Method for the 372 haploid deletion strains. The results indicate that the optimal model contains seven clusters (*k* = 7).
(TIF)

S1 Table Characteristic parameters of growth curves of deletion mutant strains assessed in liquid media. The Tables show mean values normalised with wild type, standard deviation (SD), adjusted p-value and significance per parameter.
(XLSX)

S2 Table List of transcription factors DE in the mutant strains. (XLSX)

S3 Table Genetic interaction scores between SUT126 and Pdr3. (XLSX)

S4 Table List of yeast strains and plasmids used in this study. (XLSX)

S5 Table List of primers for quantitative real time PCR (qPCR) used in this study. (XLSX)

S6 Table List of media condition used for solid fitness analysis for the haploid ncRNA deletion collection. (XLSX)
