# Supplementary material for: Functional and transcriptional profiling of non-coding RNAs in yeast reveal context-dependent phenotypes and in trans effects on the protein regulatory network
Source: PLoS Genet. 2021 Jan 25;17(1):e1008761. doi: 10.1371/journal.pgen.1008761 (PMC7886133; doi:10.1371/journal.pgen.1008761)

**Supplementary File S2.**

**Read distribution across the gene body and the 3’ UTR of the top 20 differentially expressed genes is unchanged in the ncRNA deletion strains**

Defective 3’end processing was assessed by visualising the distribution of reads across the gene body and the at the 3’ UTR of the top 20 DE genes in each mutant strain from the RNA-seq experiments. Full annotated transcripts for each of the genes are shown (annotation obtained from the Yeast Genome database project, <http://www.yeastgenome.org/>). Read density was normalized per gene to take in account the difference in expression between WT and mutant strains. To detect a potential read-trough we have extended the visualization of the reads downstream of the stop codon to include the 3’end and up until a clear drop of reads was detected (usually after 100-300 nucleotides (nt) from the stop codon of each gene). The following images show: ***i.*** comparison of the distribution of reads across the open reading frame, including the 3’ UTR, of the 20 top differential expressed genes in *SUT125Δ, SUT126Δ*, *SUT035Δ* and *SUT532Δ* mutant strains compared with the WT strain BY4741 (A); ***ii.*** comparison of the distribution of reads in the zoomed in 3’ UTR, of the 20 top differential expressed genes in the previous mentioned mutant strain compared with the WT strain BY4741 (B). Three biological replicates are shown with the normalized read density. The gene names are reported at the bottom of each panel and are represented by black or blue boxes, according to whether they are on a Watson or a Crick strand. The scale in nucleotides (1000 nt for the ORF and 500 nt for the zoomed in portion of the 3’ UTR) is represented by a black bar on the top of the panels.

(**A**): Images showing the RNA-seq reads (ORFs and 3’UTRs) comparing the wild type BY4741 with the 20 top differential expressed genes in *SUT125Δ*, *SUT126Δ, SUT035Δ* and *SUT532Δ* mutant strains.

**BY4741 vs *SUT125*Δ**

_
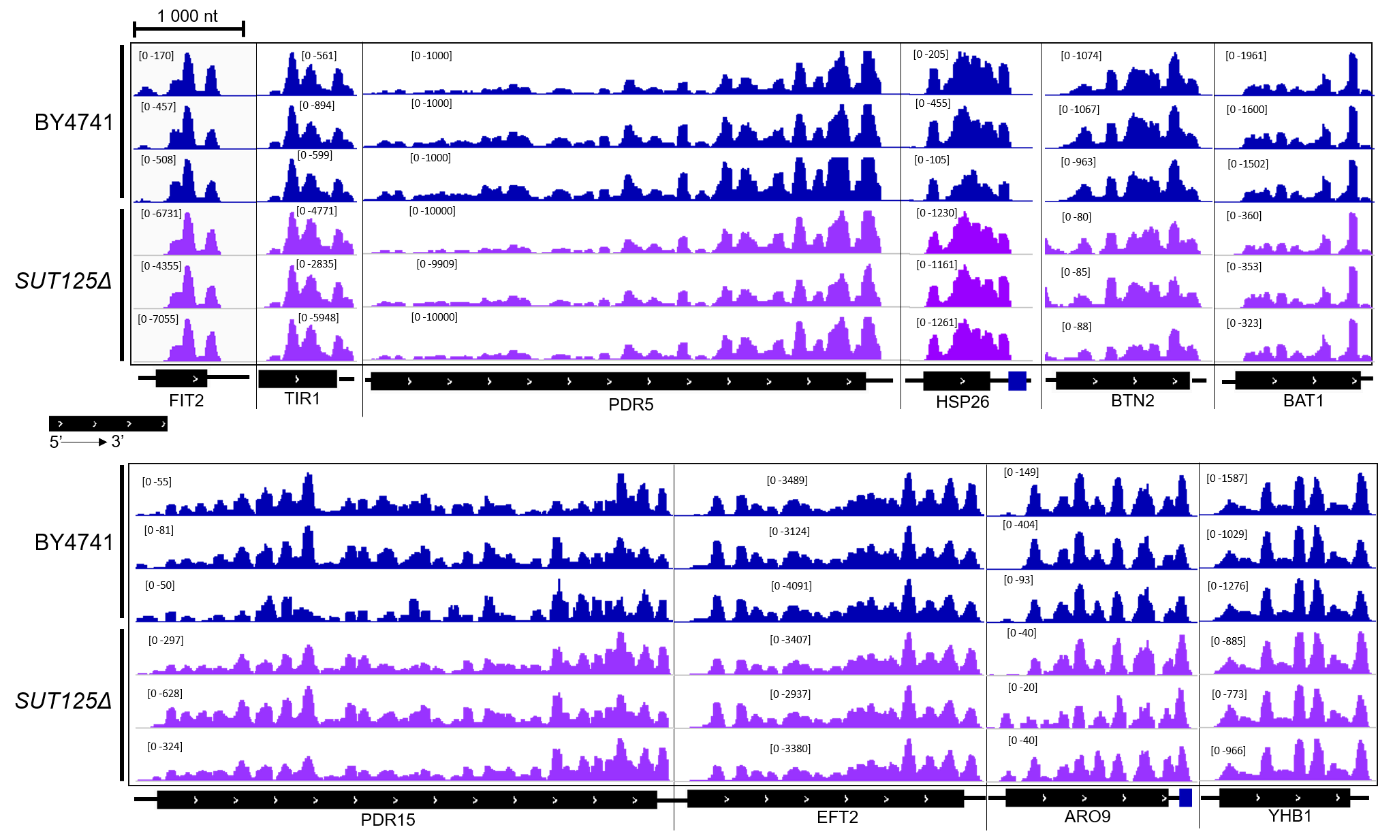
_


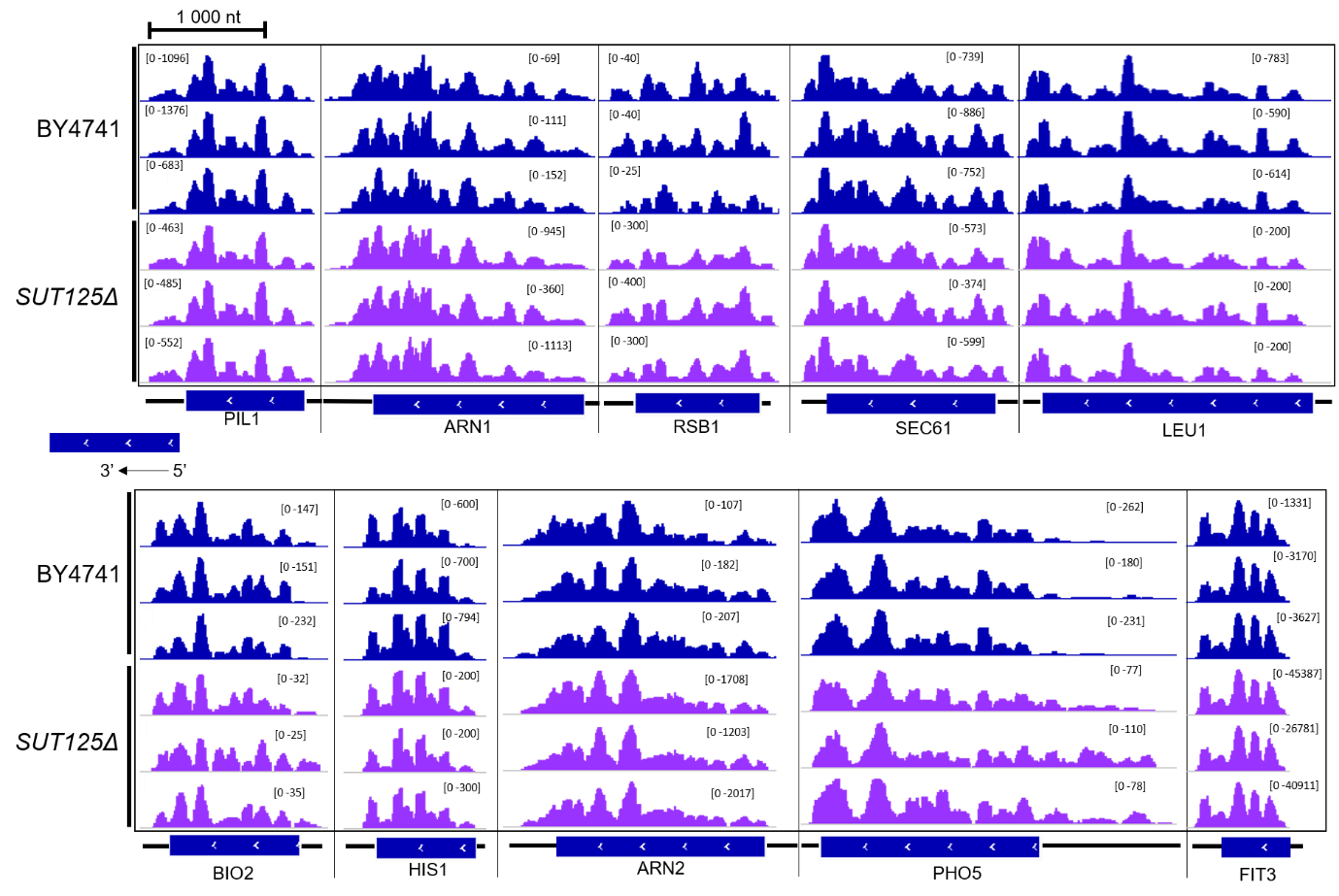


**BY4741 vs *SUT126*Δ**


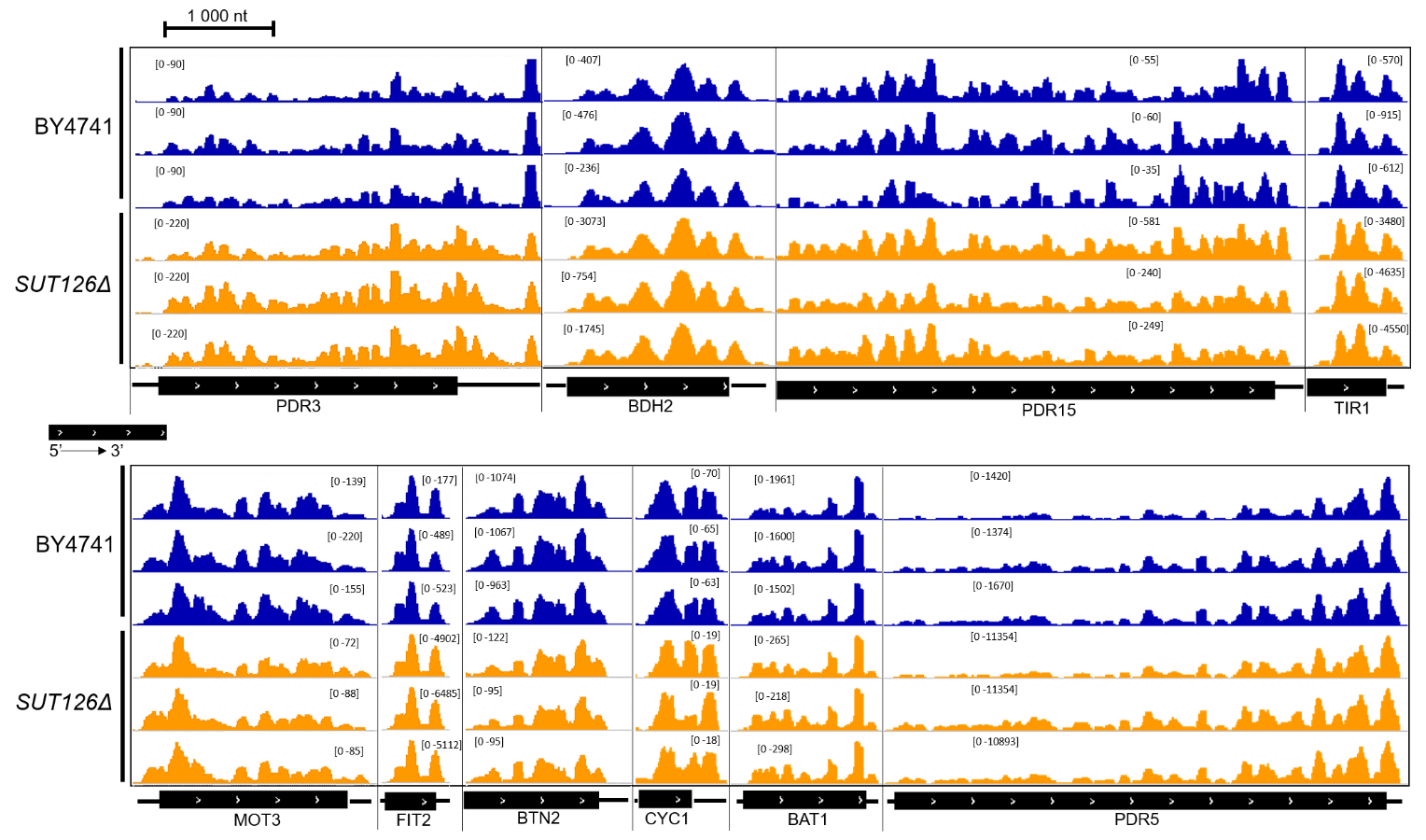

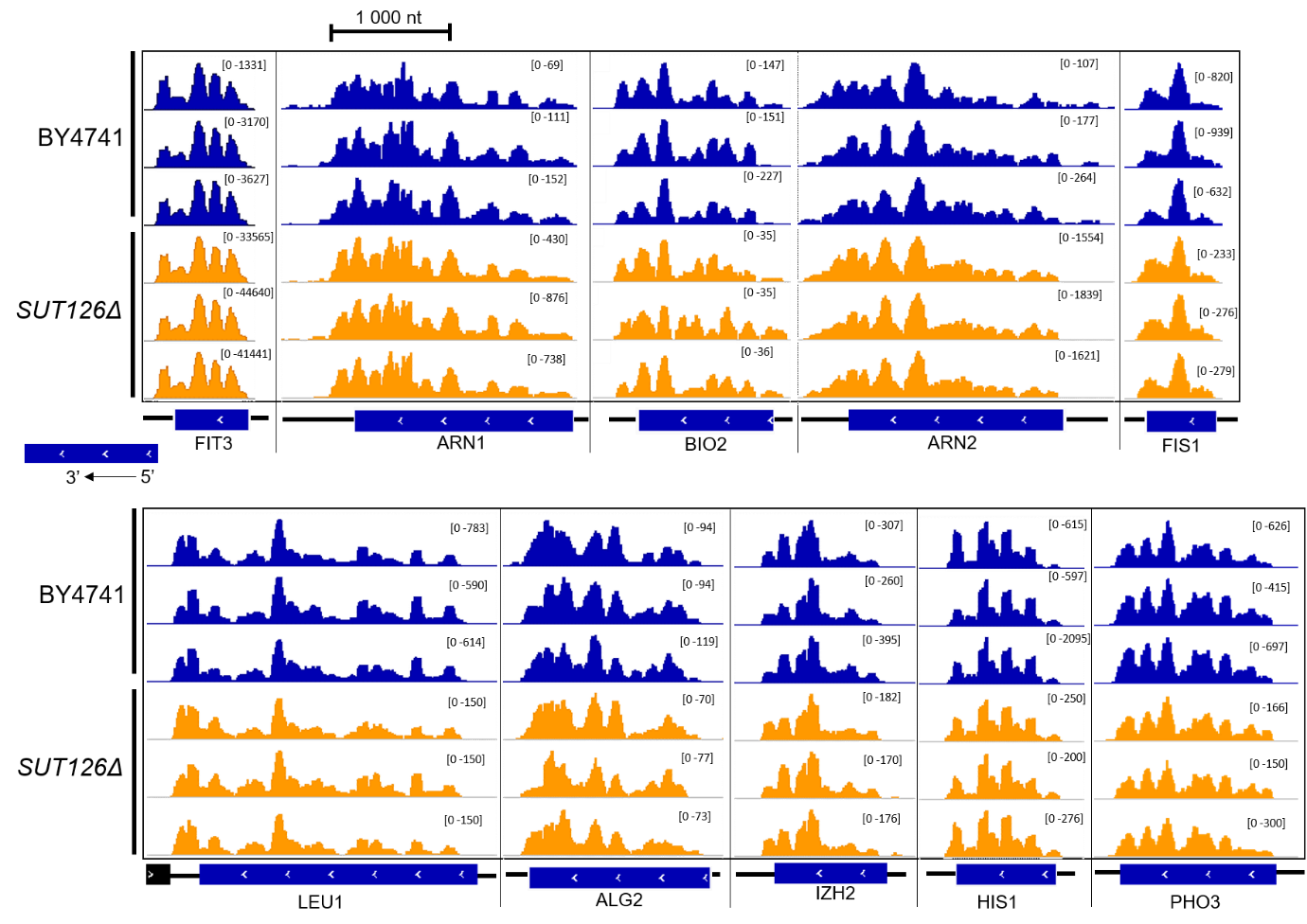


**BY4741 vs *SUT035*Δ**


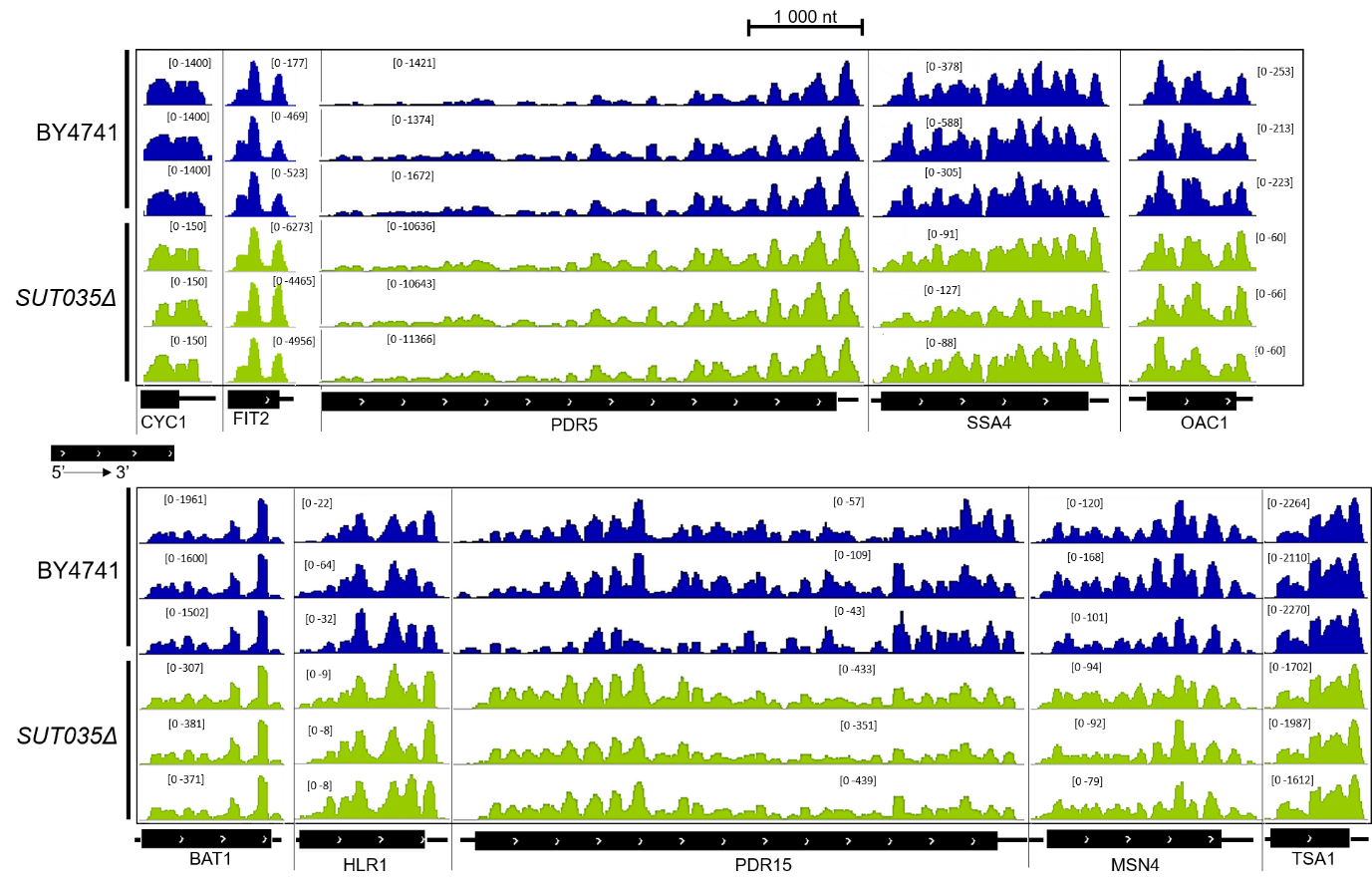

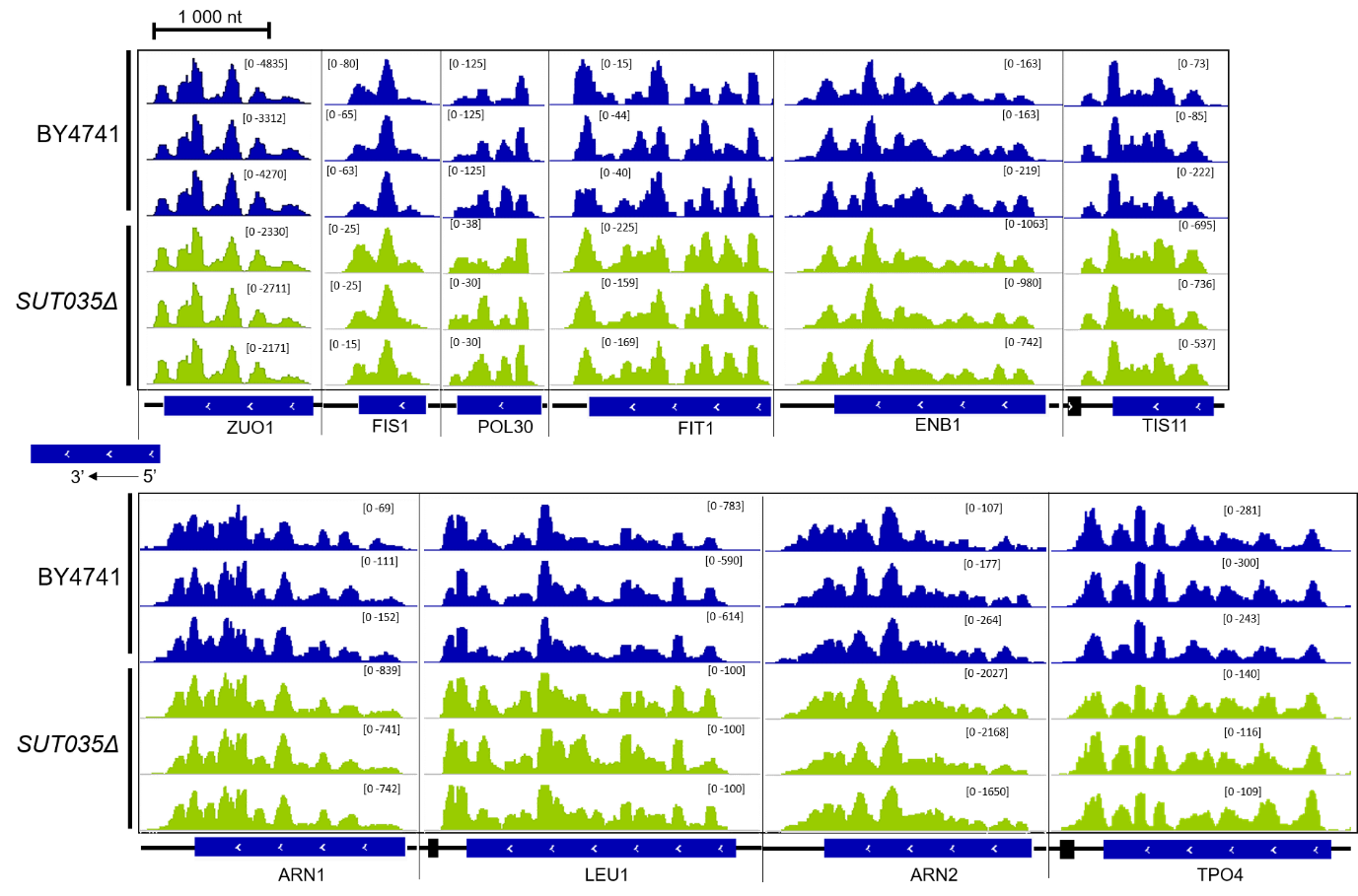


**BY4741 vs *SUT532*Δ**

**
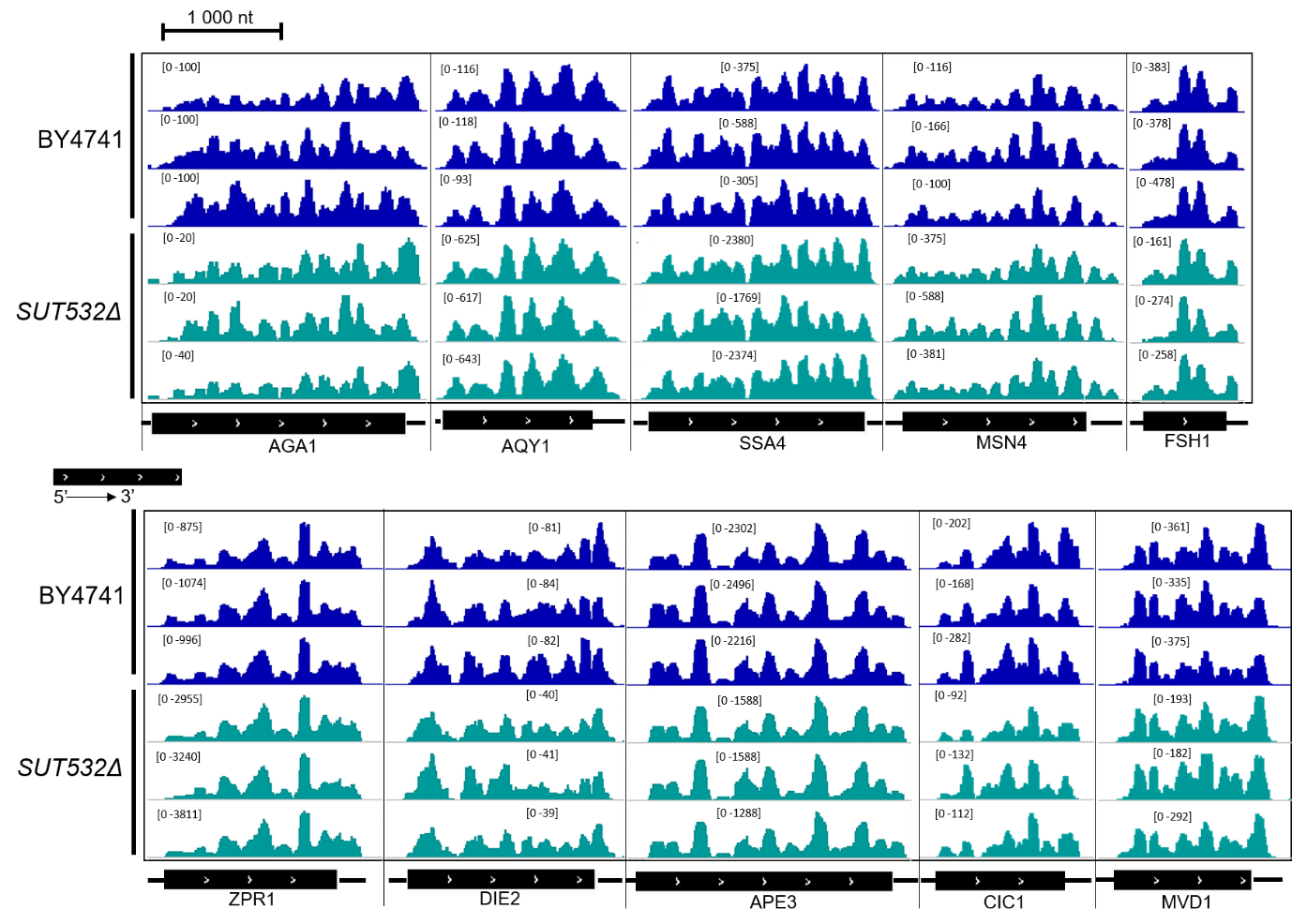

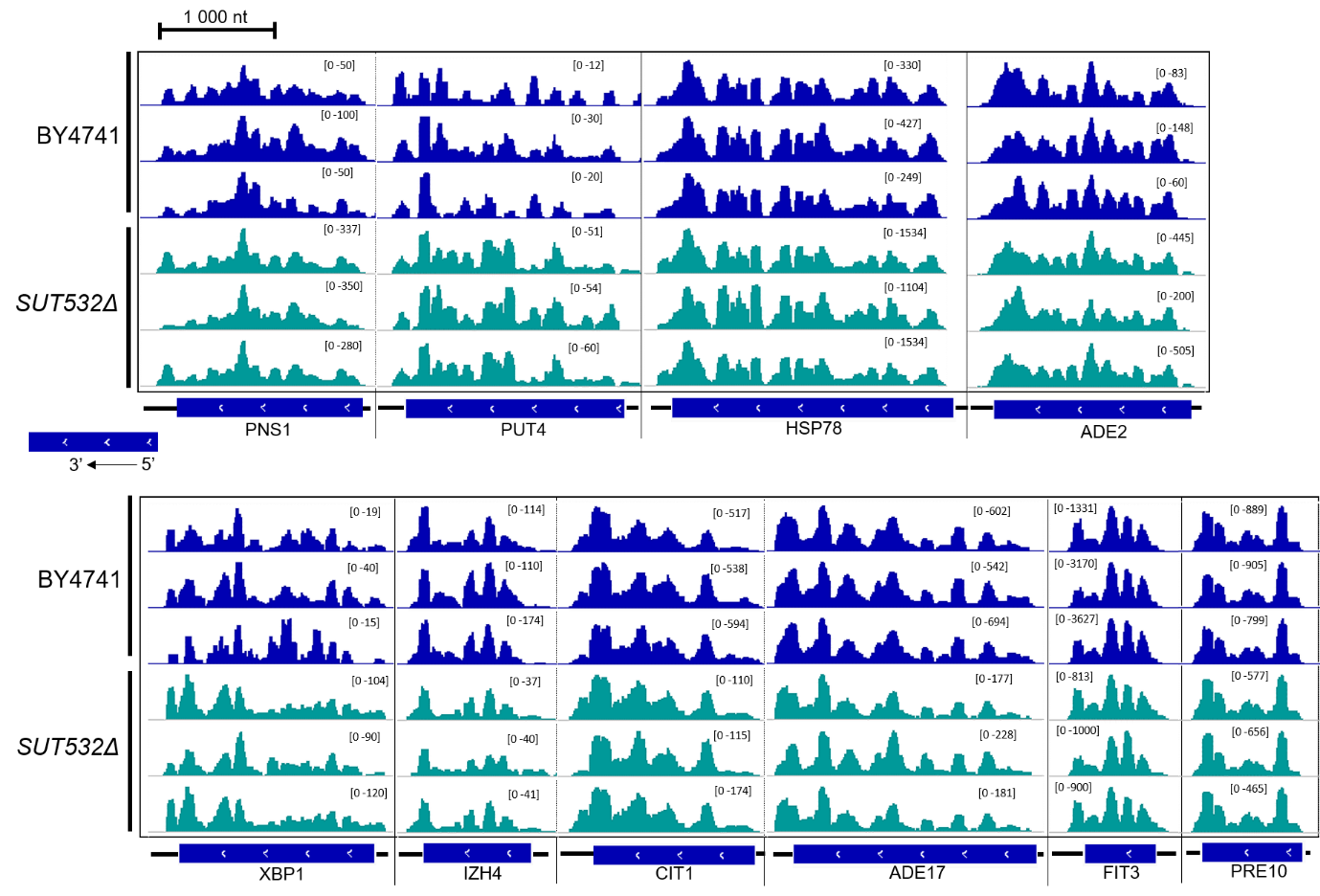
**

(**B**): Images zoomed in at 3’ UTR of the top 20 DE genes in *SUT125Δ*, *SUT126Δ, SUT035Δ* and *SUT532Δ* mutant strains. Every panel shows the 3’-end of the genes and an extension between 100 - 300 nt downstream from the 3’-end of the ORFs. The extension of reads is displayed until a complete drop on the read density was detected.

**BY4741 vs *SUT125*Δ**


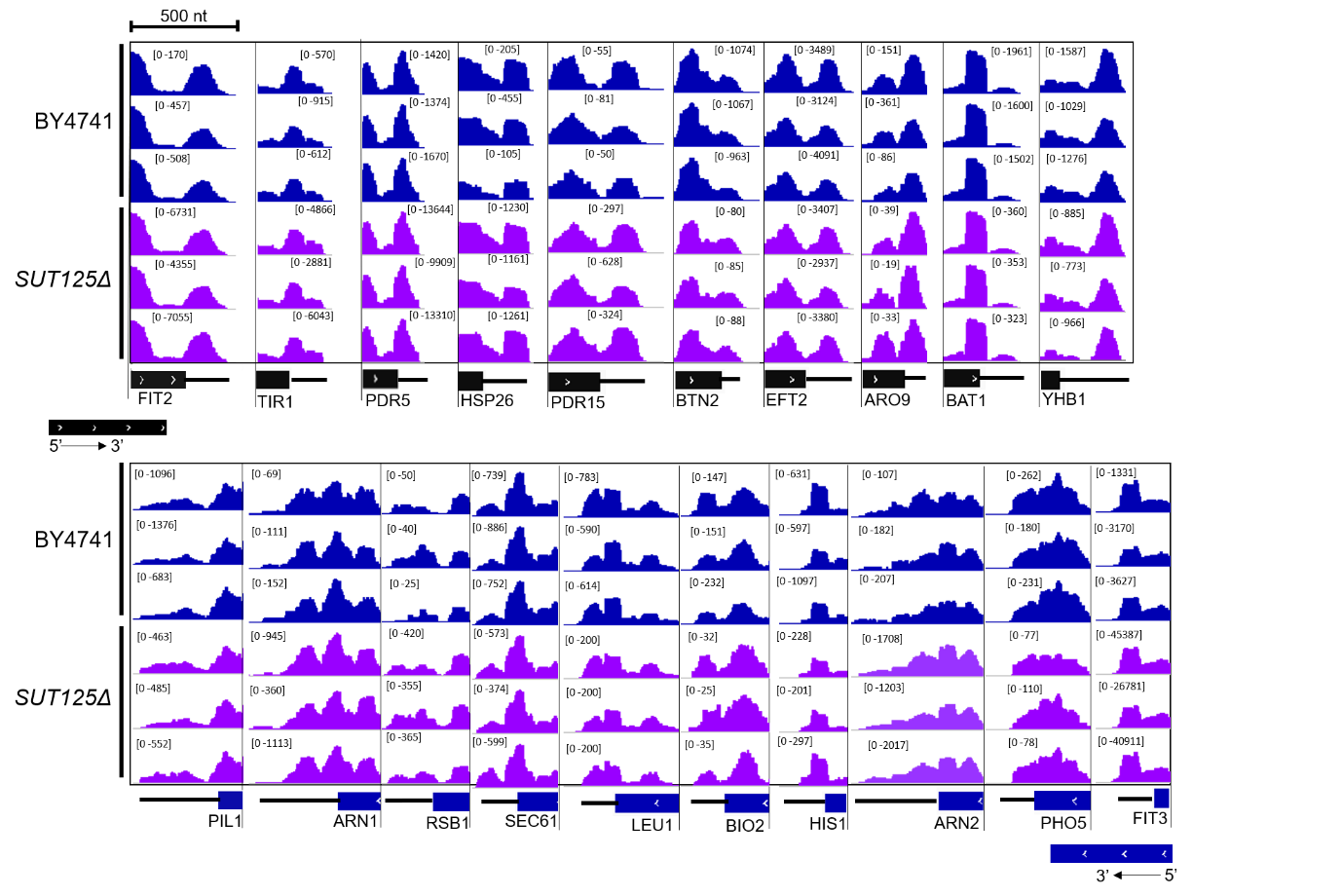


**BY4741 vs *SUT126*Δ**


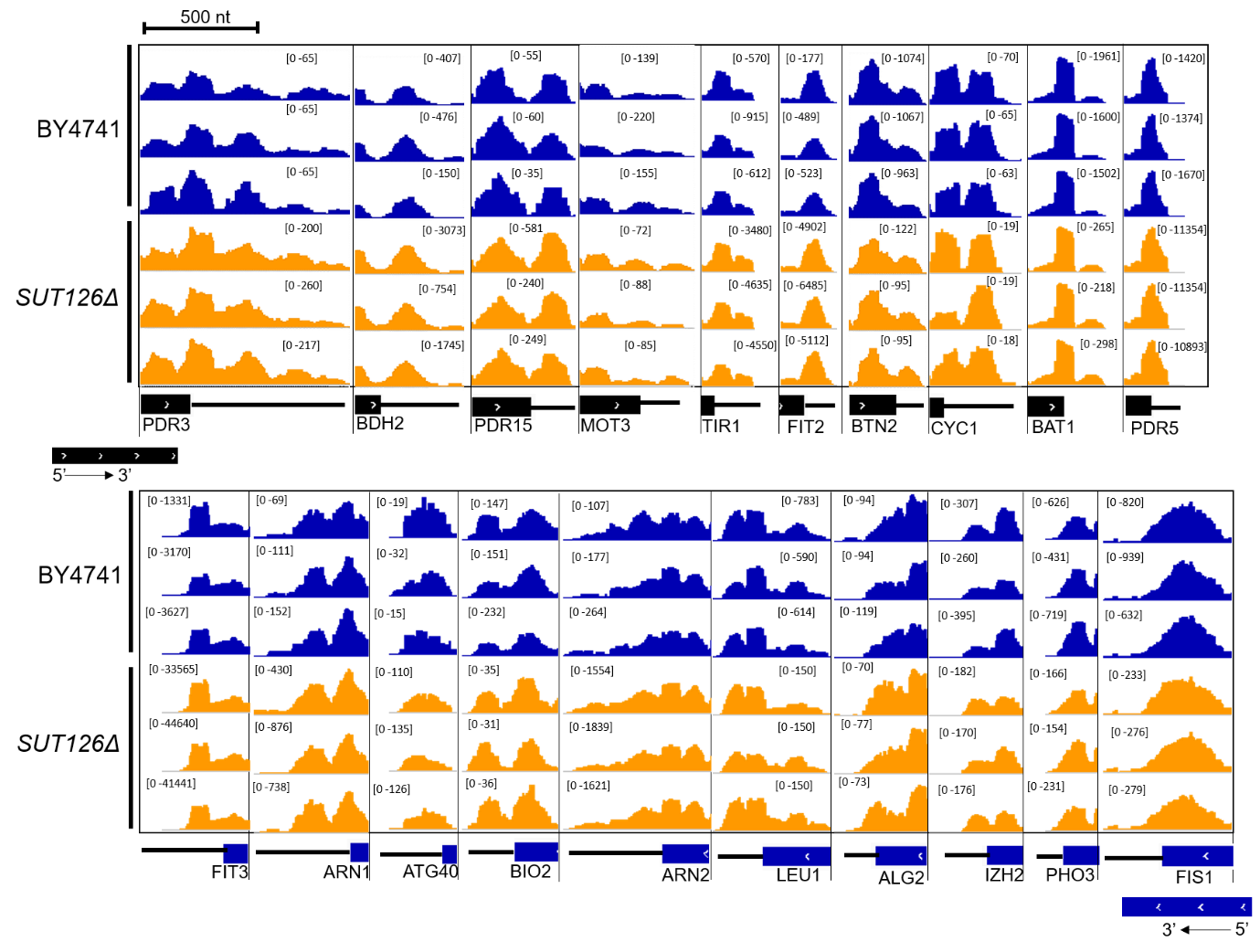


**BY4741 vs *SUT035*Δ**


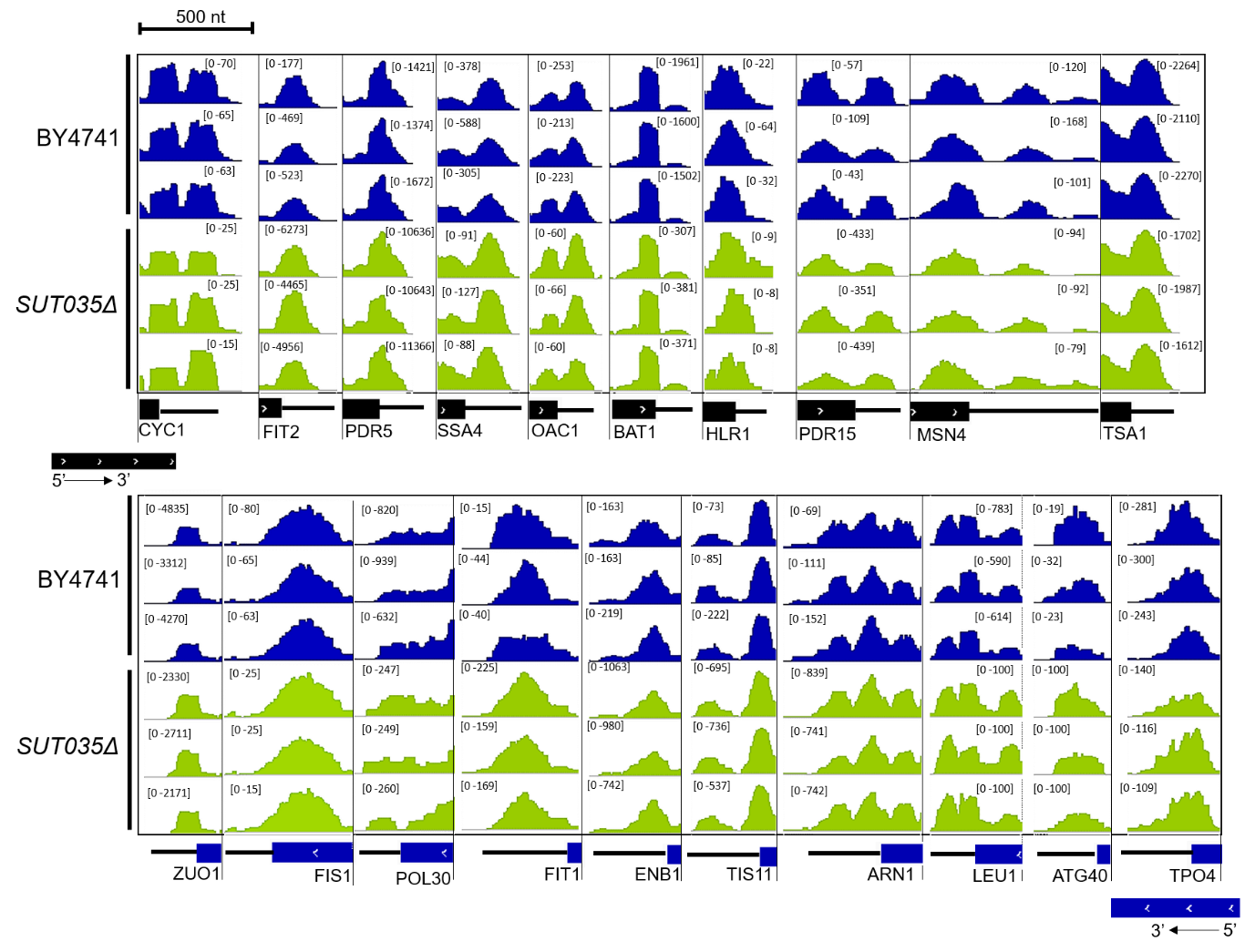


**BY4741 vs *SUT532*Δ**


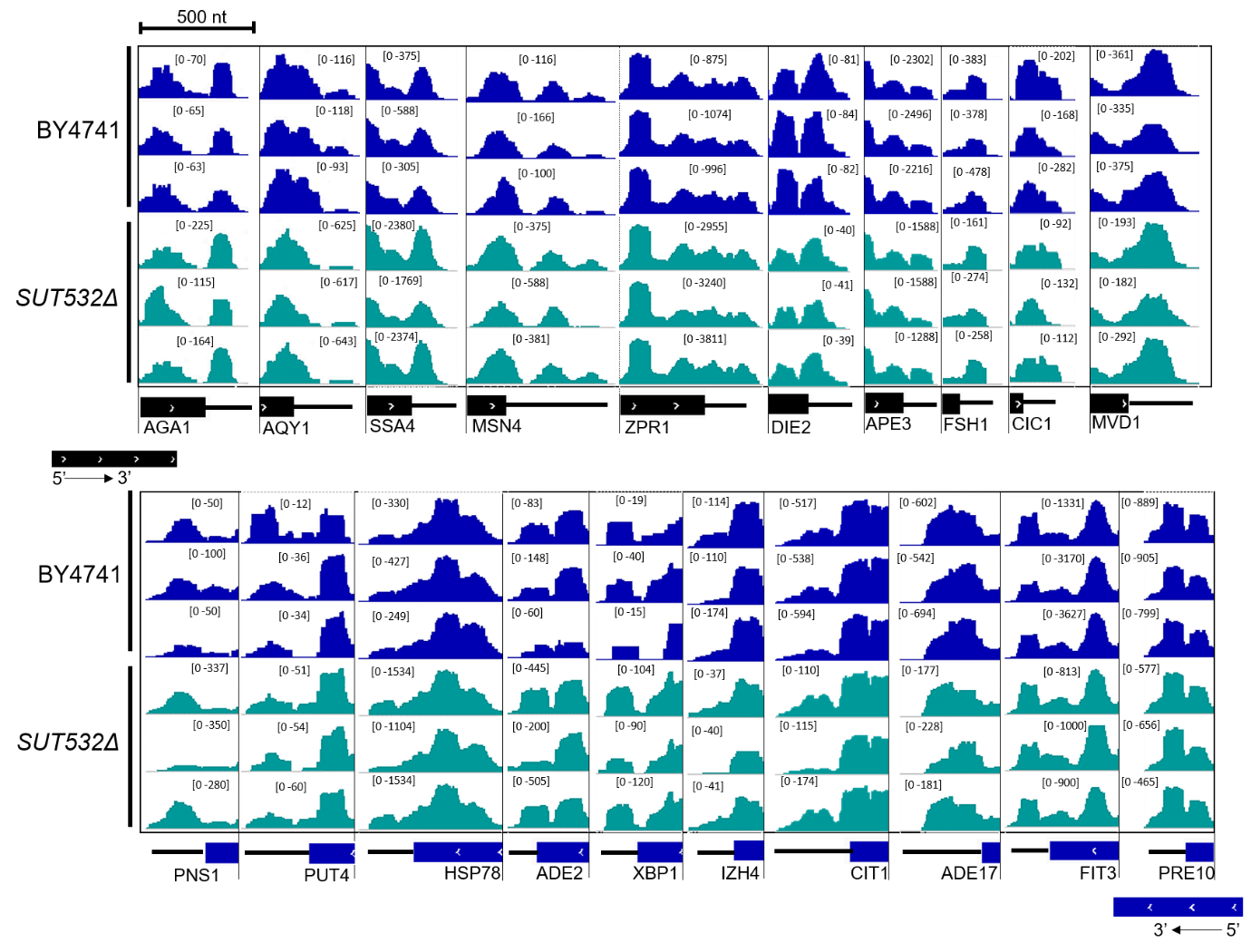

Supplement: S2 File — The entire ORF and a specific “zoom in” on the 3’UTR is visualised. (DOCX) [file pgen.1008761.s004.docx]
