# Supplementary figures and images for: Functional and transcriptional profiling of non-coding RNAs in yeast reveal context-dependent phenotypes and in trans effects on the protein regulatory network
Source: PLoS Genet. 2021 Jan 25;17(1):e1008761. doi: 10.1371/journal.pgen.1008761 (PMC7886133; doi:10.1371/journal.pgen.1008761)

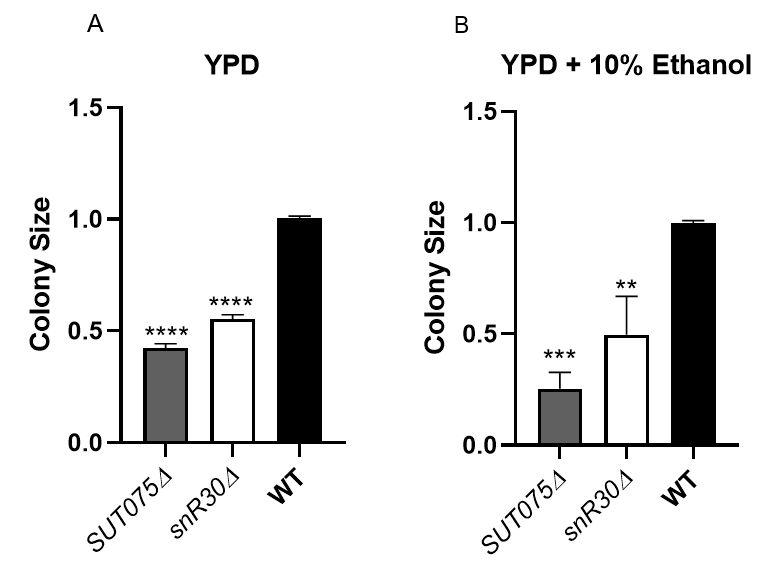

Supplement: S1 Fig — Bar charts displays the colony size of SUT075Δ and snR30Δ deletion strains when growing in (A) YPD and (B) YPD supplemented with 10% ethanol. (TIF) [file pgen.1008761.s005.tif]

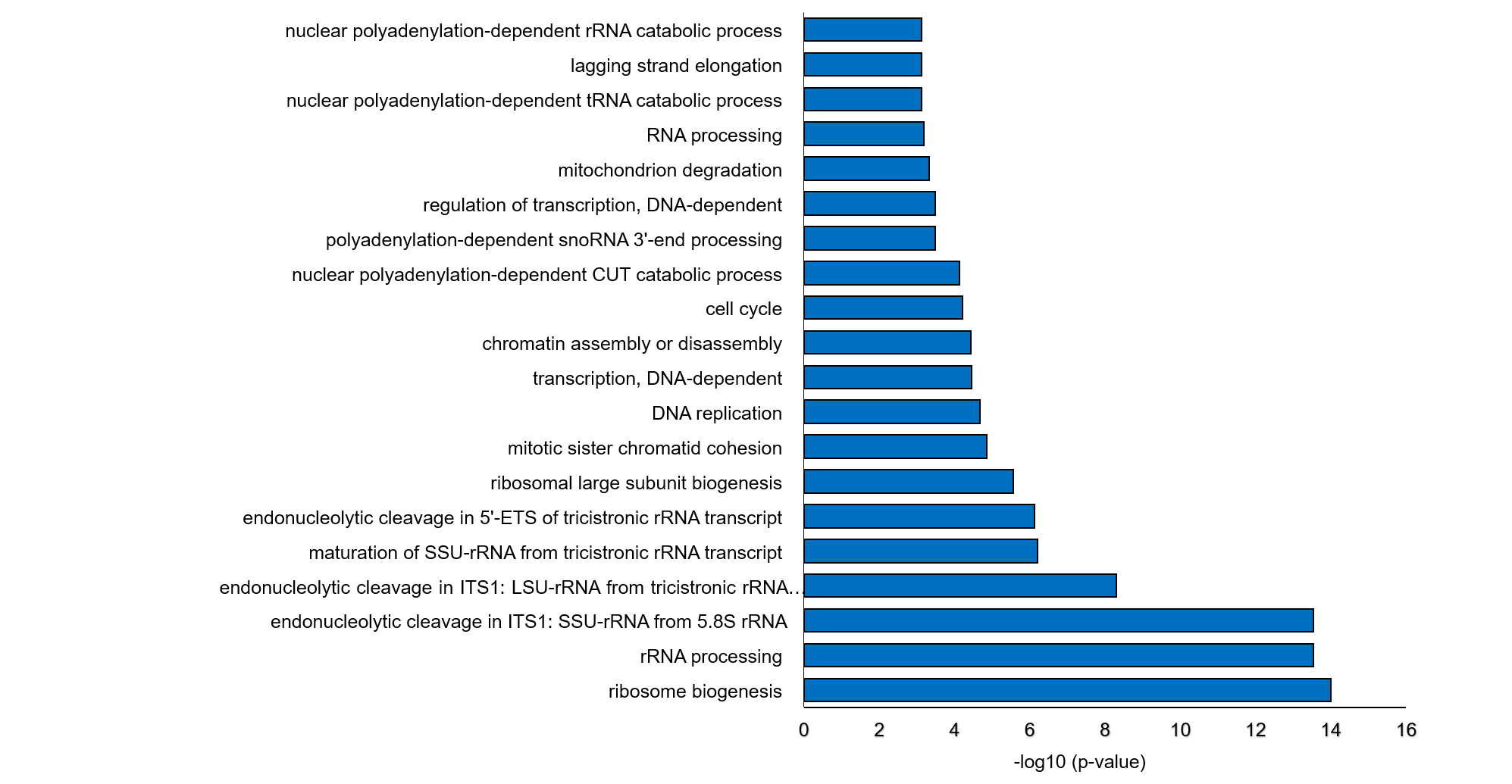

Supplement: S2 Fig — Bar chart displaying the top 20 significantly enriched GO terms. The negative logarithm of the adjusted p-value (base 10) after Holm-Bonferroni correction is represented on the x-axis. The figure was created using the DE genes in common for SUT075 and snR30 deletion mutants (n = 1836). (TIF) [file pgen.1008761.s006.tif]

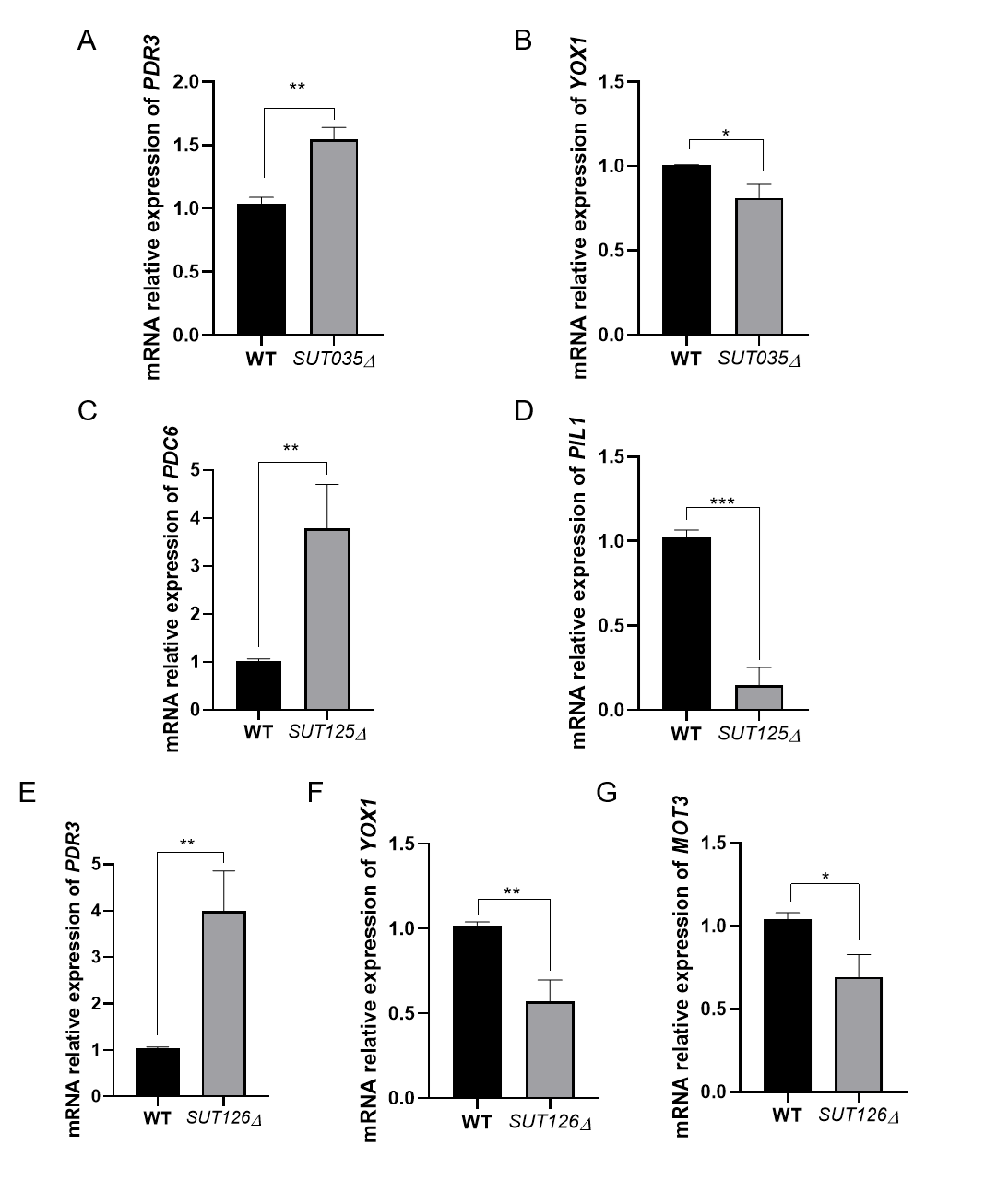

Supplement: S3 Fig — Relative mRNA levels of (A) PDR3 and (B) YOX1 in SUT035Δ strain, (C) PDC6 and (D) PIL1 in SUT125Δ and the TFs (E) PDR3, (F) YOX1 and (G) MOT3 in SUT126Δ strain analysed by RT-qPCR. Relative mRNA levels were quantified by qPCR and compared by t-test. (TIF) [file pgen.1008761.s007.tif]

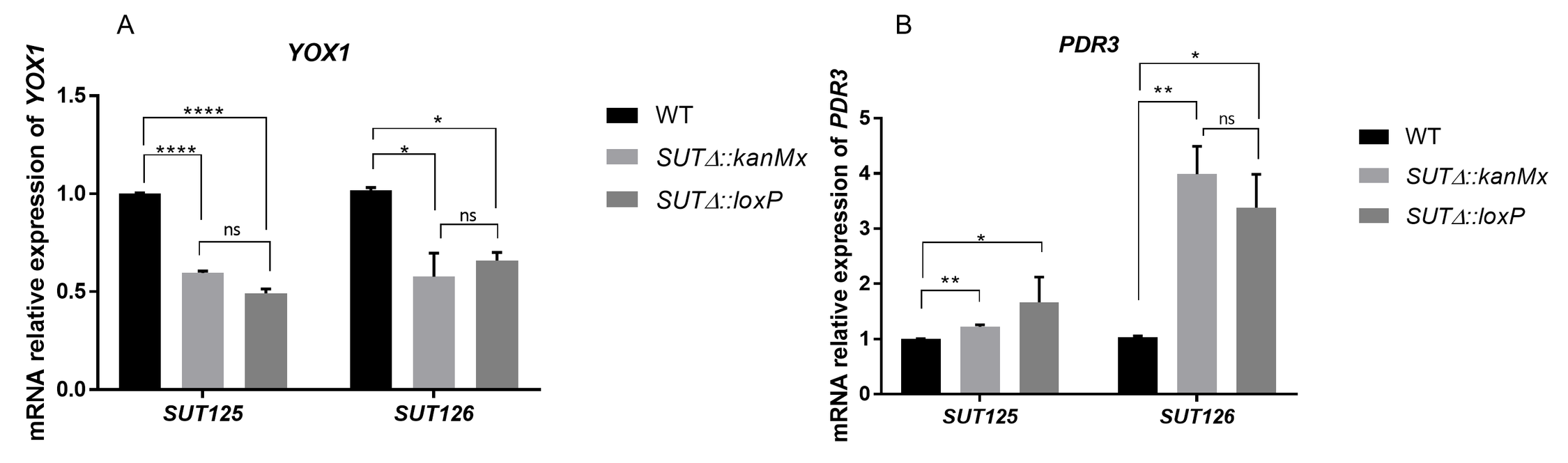

Supplement: S4 Fig — Relative mRNA levels of the transcriptional repressor (A) YOX1 and the transcriptional activator (B) PDR3 in SUT125Δ and SUT126Δ deletion mutant strains with and without kanMX. The kanMX cassette does not influence genes located distantly from the SUT disruption. Relative mRNA levels were quantified by qPCR and compared by ANOVA. (TIF) [file pgen.1008761.s008.tif]

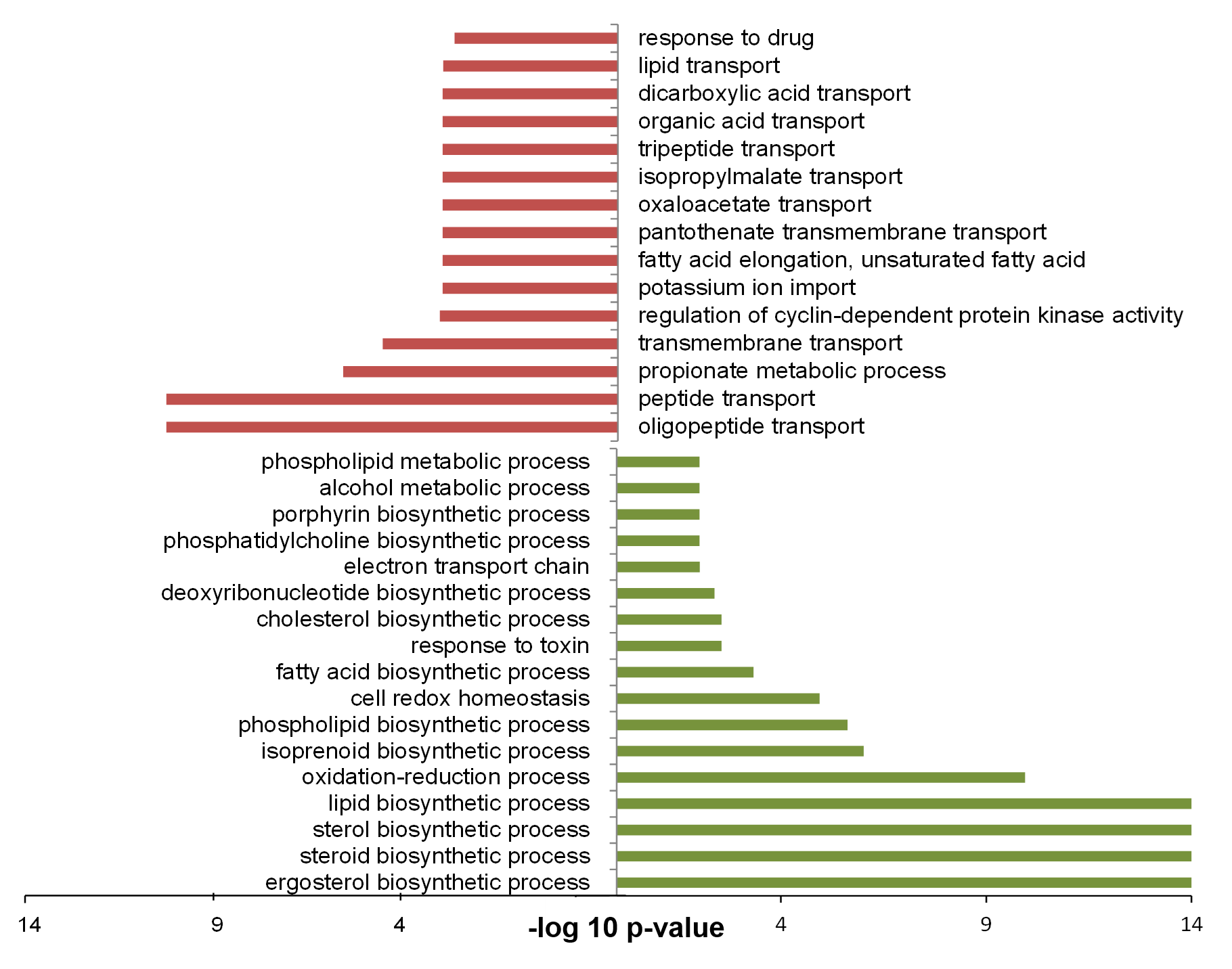

Supplement: S5 Fig — Representative GO terms for biological processes for up-regulated (red) and down- regulated (green) genes in the CUT494/SUT053/SUT468Δ strain. The p-value cutoff after Holm-Bonferroni correction is < 0.05; y–axis displays GO terms, x-axis shows the p-value that was transformed to–log10. The figure was created using the DE genes (n = 137). (TIF) [file pgen.1008761.s009.tif]

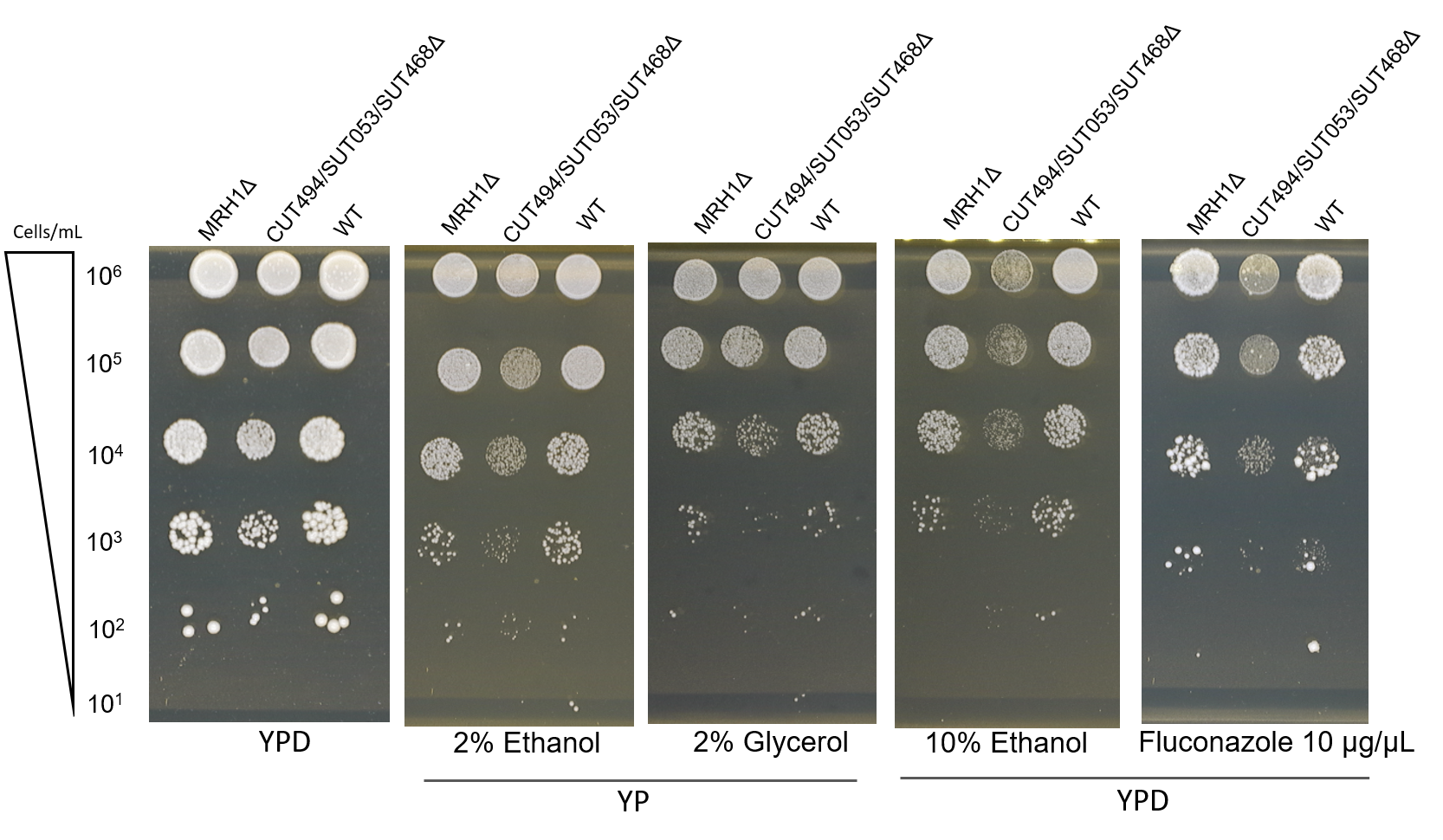

Supplement: S6 Fig — The growth of CUT494/SUT053/SUT468Δ, MRH1Δ and WT strains were analysed via spot tests on YPD, YPD + 10% ethanol, YP + 2% glycerol, YP + 2% ethanol and YPD+ 10μg/μL fluconazole. MRH1 is an integral component of the membrane, downregulated in CUT494/SUT053/SUT468Δ mutant strains, and located nearby. The fitness impairment detected in CUT494/SUT053/SUT468Δ strain under stress conditions is independent of its effect on the neighboring gene MRH1. (TIF) [file pgen.1008761.s010.tif]

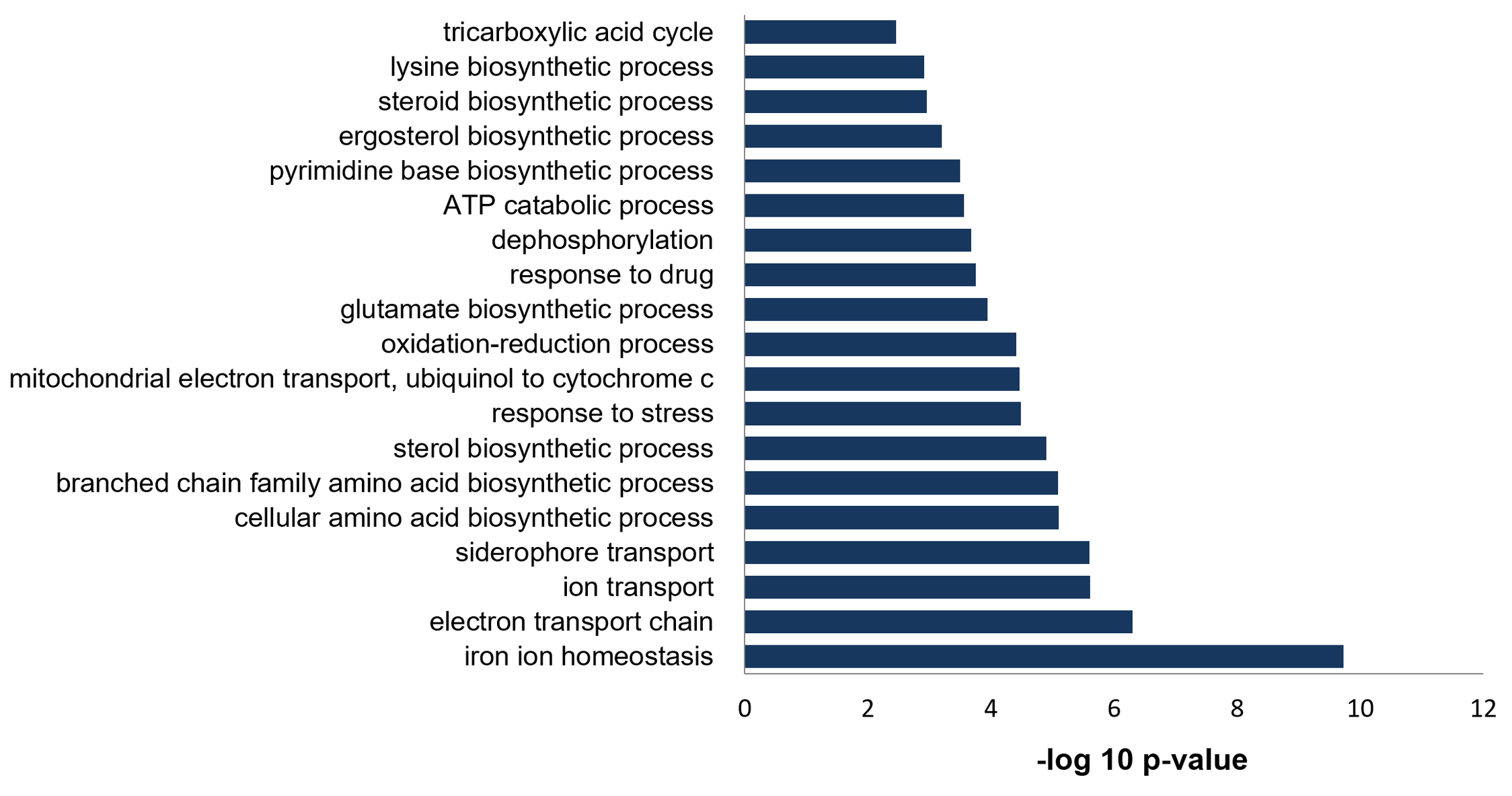

Supplement: S7 Fig — Gene Ontology of biological processes inferred from dysregulated coding targets in common in SUT125Δ, SUT126Δ and SUT035Δ deletion strains. Significantly first enriched GO terms for biological processes (Holm-Bonferroni adjusted p-value <0.05) are listed on the y-axis, and the negative log of the adjusted p-value (base 10) is represented on the x-axis. The figure was created using the DE genes in common for SUT125, SUT126 and SUT035 (n = 481). (TIF) [file pgen.1008761.s011.tif]

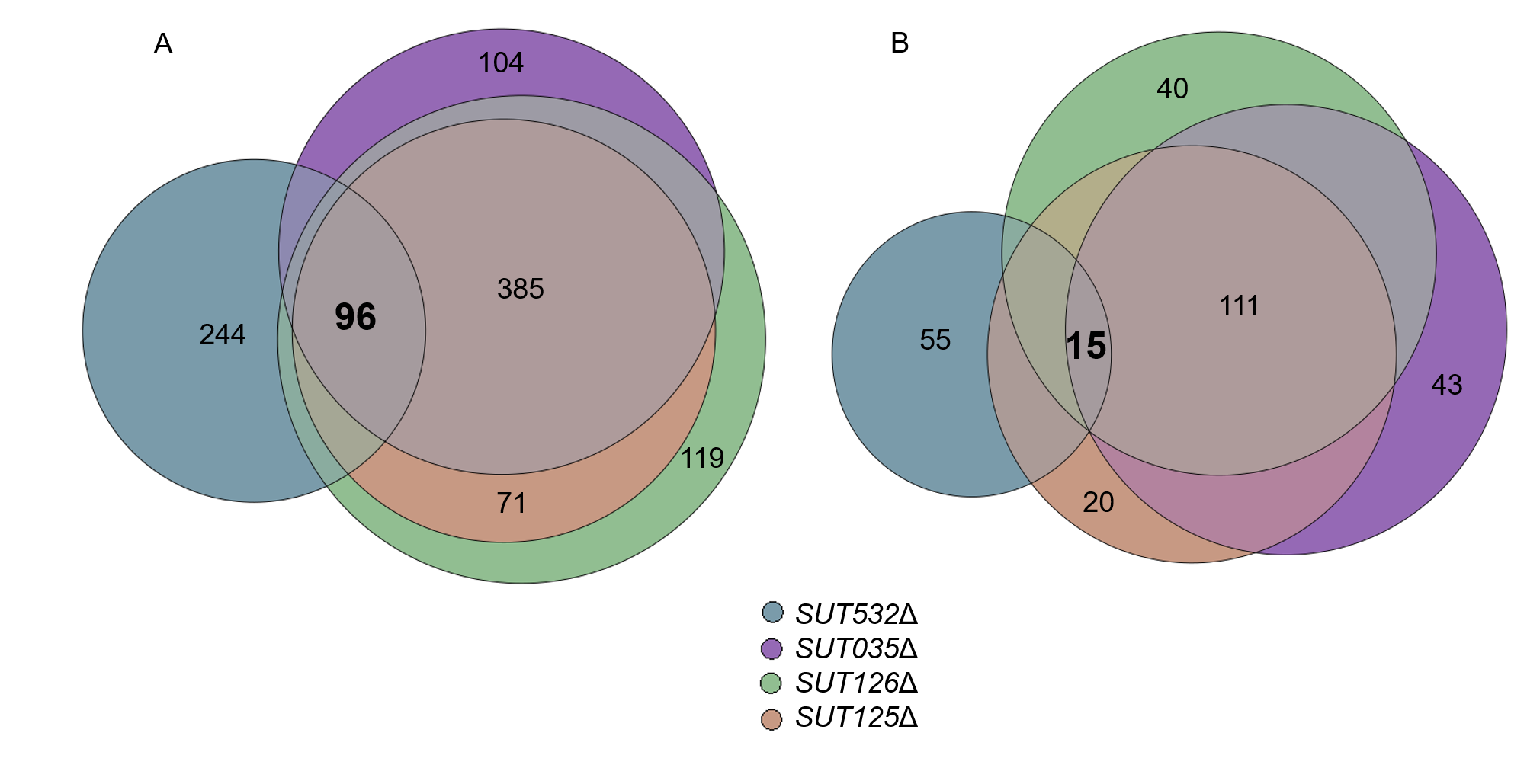

Supplement: S8 Fig — Number of (A) protein coding genes (96) and (B) non-coding transcripts (15) in common dysregulated among SUT125Δ, SUT035Δ, SUT126Δ and SUT532Δ deletion strains. Venn diagram was generated using Eulerr [91]. (TIF) [file pgen.1008761.s012.tif]

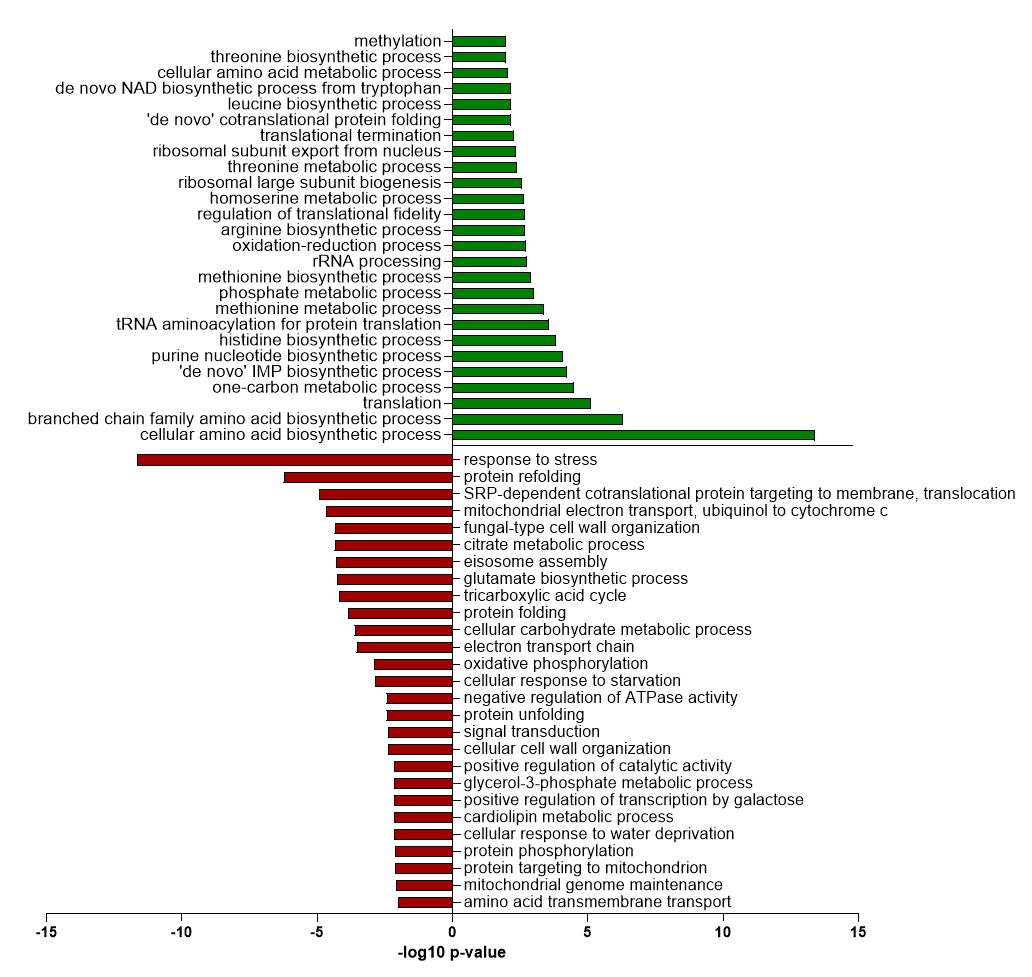

Supplement: S9 Fig — Significantly enriched representative GO terms for biological processes for up-regulated (red, n = 172) and down-regulated (green, n = 236) in SUT532Δ deletion strain. P-value was calculated using Holm-Bonferroni correction. Representative GO terms are listed on the y-axis, and the negative log of the adjusted p-value (base 10) is represented on the x-axis. (TIF) [file pgen.1008761.s013.tif]

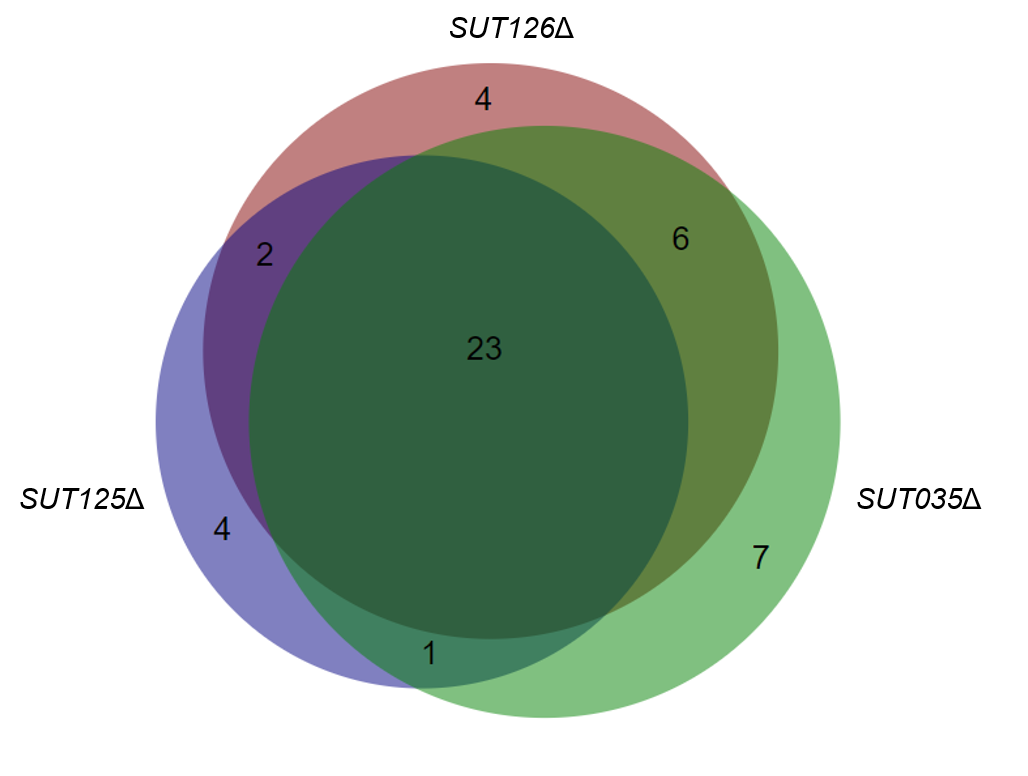

Supplement: S10 Fig — Area proportional Venn diagram generated by BioVenn [71] using the number of TFs dysregulated in deletion strains in cluster 1 (SUT125Δ, SUT035Δ) and 2 (SUT126Δ). The overlapping (23 TFs) is shown in a dark green colour. (TIF) [file pgen.1008761.s014.tif]

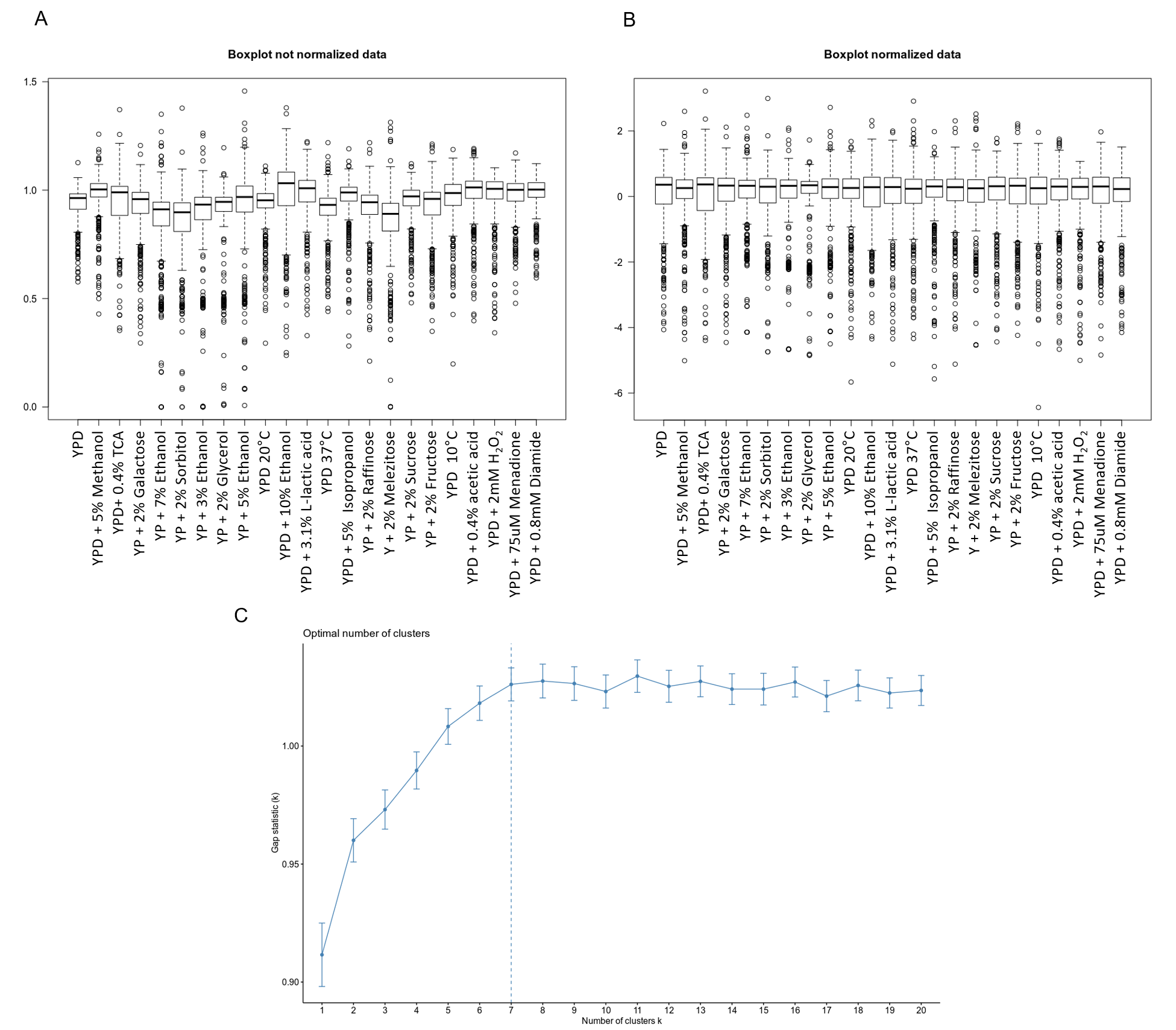

Supplement: S11 Fig — Representative box plot of raw (A) and normalized (B) data from three technical replicates of colony size for 372 ncRNAs deletion strains in 23 different environmental conditions. The box plot indicates median-centered raw data distributions of the fitness per condition tested, which were further refined during normalisation. The horizontal axis stands for the media, while the vertical axis represents the colony sizes. (C) Optimal number of clusters were calculated by the Gap Statistic Method for the 372 haploid deletion strains. The results indicate that the optimal model contains seven clusters (k = 7). (TIF) [file pgen.1008761.s015.tif]
